# Long noncoding RNA ZFAS1 promoting small nucleolar RNA-mediated 2′-O-methylation via NOP58 recruitment in colorectal cancer

**DOI:** 10.1186/s12943-020-01201-w

**Published:** 2020-05-22

**Authors:** Huizhe Wu, Wenyan Qin, Senxu Lu, Xiufang Wang, Jing Zhang, Tong Sun, Xiaoyun Hu, Yalun Li, Qiuchen Chen, Yuanhe Wang, Haishan Zhao, Haiyan Piao, Rui Zhang, Minjie Wei

**Affiliations:** 1 Department of Pharmacology, School of Pharmacy, China Medical University, Shenyang, 110122 People’s Republic of China; 2 Liaoning Key Laboratory of molecular targeted anti-tumor drug development and evaluation; Liaoning Cancer immune peptide drug Engineering Technology Research Center; Key Laboratory of Precision Diagnosis and Treatment of Gastrointestinal Tumors, Ministry of Education, China Medical University, Shenyang, 110122 People’s Republic of China; 3 Department of Anorectal Surgery, First Hospital of China Medical University, Shenyang, 110001 People’s Republic of China; 4 Department of Medical Oncology, Cancer Hospital of China Medical University, Shenyang, 110042 People’s Republic of China

**Keywords:** ZFAS1, SNORD12C, SNORD78, 2′-O-methylation (2′-O-me), NOP58, Colorectal cancer (CRC)

## Abstract

**Background:**

Increasing evidence supports the role of small nucleolar RNAs (snoRNAs) and long non-coding RNAs (lncRNAs) as master gene regulators at the epigenetic modification level. However, the underlying mechanism of these functional ncRNAs in colorectal cancer (CRC) has not been well investigated.

**Methods:**

The dysregulated expression profiling of lncRNAs-snoRNAs-mRNAs and their correlations and co-expression enrichment were assessed by *GeneChip* microarray analysis. The candidate lncRNAs, snoRNAs, and target genes were detected by in situ hybridization (ISH), RT-PCR, qPCR and immunofluorescence (IF) assays. The biological functions of these factors were investigated using in vitro and in vivo studies that included CCK8, trans-well, cell apoptosis, IF assay, western blot method, and the xenograft mice models. rRNA 2′-O-methylation (Me) activities were determined by the RTL-P assay and a novel double-stranded primer based on the single-stranded toehold (DPBST) assay. The underlying molecular mechanisms were explored by bioinformatics and RNA stability, RNA fluorescence ISH, RNA pull-down and translation inhibition assays.

**Results:**

To demonstrate the involvement of lncRNA and snoRNAs in 2′-O-Me modification during tumorigenesis, we uncovered a previously unreported mechanism linking the snoRNPs NOP58 regulated by ZFAS1 in control of SNORD12C, SNORD78 mediated rRNA 2′-O-Me activities in CRC initiation and development. Specifically, ZFAS1 exerts its oncogenic functions and significantly up-regulated accompanied by elevated NOP58, SNORD12C/78 expression in CRC cells and tissues. ZFAS1 knockdown suppressed CRC cell proliferation, migration, and increased cell apoptosis, and this inhibitory effect could be reversed by NOP58 overexpression in vitro and in vivo. Mechanistically, the NOP58 protein could be recognized by the specific motif (AAGA or CAGA) of ZFAS1. This event accelerates the assembly of SNORD12C/78 to allow for further guiding of 2′-O-Me at the corresponding Gm3878 and Gm4593 sites. Importantly, silencing SNORD12C or 78 reduced the rRNAs 2′-O-Me activities, which could be rescued by overexpression ZFAS1, and this subsequently inhibits the RNA stability and translation activity of their downstream targets (e.g., EIF4A3 and LAMC2).

**Conclusion:**

The novel ZFAS1-NOP58-SNORD12C/78-EIF4A3/LAMC2 signaling axis that functions in CRC tumorigenesis provides a better understanding regarding the role of lncRNA-snoRNP-mediated rRNAs 2′-O-Me activities for the prevention and treatment of CRC.

## Introduction

Recent evidence has demonstrated that non-coding RNAs (ncRNAs) such as small nucleolar RNAs (snoRNAs) and long non-coding RNAs (lncRNAs) can act as master gene regulators at the transcriptional and post-transcriptional epigenetic levels, and aberrant functions of these RNAs are established hallmarks of tumorigenesis [1, 2]. Notably, a number of RNA modifications, including ribosome RNA (rRNA) 2′-O-methylation (2′-O-Me), play an essential role in the regulation of gene expression by altering and fine-tuning the properties of mRNAs, rRNAs and lncRNAs [3–5]. Specifically, the most clearly understood function of the C/D box small nucleolar RNAs (SNORDs) is their ability to assemble small nucleolar ribonucleoprotein complexes (snoRNPs) comprised of core RNP proteins (NOP58, NOP56, SNU13, FBL) and rRNA such as SNORD27, which facilitates 2′-O-Me of A27 on 18S rRNA [6, 7]. The suppression of snoRNAs specific to U26, U44, and U78 (corresponding to 28S-Am398, 18S-Am163, and 28S-Gm3745) reduced rRNA modifications at the corresponding sites and led to severe morphological defects and embryonic lethality, suggesting a critical role for these rRNA 2′-O-Me events in vertebrate development [8]. The endogenous mechanism responsible for regulating these snoRNAs and their ability to mediate the modification-based regulatory network, however, has not yet been thoroughly investigated in the context of human tumorigenesis. Thus, it is crucial to clarify the expression regulation pattern and the host gene lncRNAs and snoRNAs involved in the progression and development of solid tumors.

Despite the discovery of snoRNAs in multiple species ranging from bacteria to mammals in the 60’s, the functional role of these molecules has remained enigmatic, and no cellular functions have been identified until recently [9, 10]. Recent studies have revealed the significance and characteristics of snoRNAs in the context of tumorigenesis. For example, overexpression of SNORD78 (C/D box) was observed in non-small cell lung cancer and in hepatocellular carcinoma [11, 12]. Additionally, SNORD50A/B (C/D box) is deleted by directly binding to Kras, and then affecting Kras expression across multiple types of cancers [13]. Increased SNORA42 (H/ACA box) expression also serves as an independent prognostic factor for overall survival times among cancer patients [14]. SNORA55 (H/ACA box) silencing in prostate cancer cell lines significantly inhibits cell proliferation and migration [15]. These findings highlight the potential roles of snoRNAs in tumorigenesis, regardless of their C/D box or H/ACA box classification. Despite the emerging knowledge regarding the roles of snoRNAs in cancer, the expression landscape, regulation network, and clinical relevance of snoRNAs have not been systematically investigated in regard to cancer. These novel functions require further clarification.

Of note, a number of the guide snoRNA-hosting genes in humans are spliced, polyadenylated lncRNAs that are dynamically regulated during cell proliferation, differentiation, and apoptosis [16–18]. These genes also exhibit cell- and tissue-specific expression patterns [19–21]. snoRNA host genes are believed to be required for the specific recruitment of snoRNPs that influence modification and eventually re-enforce cell fate decisions to ensure a step-wise developmental transition [22]. For example, the host gene lncRNA ZFAS1, which encodes three C/D box SNORD12 family members (SNORD12, SNORD12B, SNORD12C), was observed to be significantly overexpressed in a variety of human malignancies such as colorectal cancer, hepatic cancer, and gastric cancer, etc. [23–27]. Based on this information, it is likely that host gene lncRNAs play critical roles in tumorigenesis and in clinical outcome. More importantly, most of the snoRNAs appear to be the similar cellular localization with their host genes and the snoRNPs complex, which suggests the possibility of synergistically regulation function in tumorigenesis. The snoRNPs assemble processes mainly include the ribosome biogenesis, modification, and maturation, events that ultimately affect protein translation fidelity [28–31]. Among these RNPs, NOP58 is an adaptor of snoRNPs that binds to the conserved C box (RUGAUGA) and D box (CUGA) of C/D box snoRNAs to provide a skeleton and bridge for the entire snoRNP complex, ultimately maintaining the homeostasis of epigenetic modifications [5]. It must be noted, however, that concrete examples of snoRNA and host gene co-regulation in response to stimuli have not yet been reported. This prompted us to examine how ribosome biogenesis and fine-tuning modification are controlled and to determine whether and how this process is involved in the recruitment of lncRNAs and snoRNPs.

In our current study, we demonstrate that the key motif of ZFAS1 directly interacts with the core component of snoRNPs/NOP58, promotes NOP58 recruitment, and accelerates SNORD12C and SNORD78 snoRNPs assembly to allow for the guiding of 2′-O-Me of 28S rRNA to specific sites (Gm3878, Gm4593). Specifically, ZFAS1 knockdown results in decreased RNA stabilization of NOP58, SNORD12C/78, and their 2′-O-Me modification guidance, and this subsequently inhibits colorectal cancer (CRC) cell proliferation and invasion and promotes cell apoptosis in vitro and in vivo. Strikingly, SNORD12C or SNORD78 inhibition decreased 2′-O-Me modification regulated by ZFAS1, and this inhibited the RNA stability and translation activity of their downstream targets such as EIF4A3, LAMC2, and others. Based on this, we identified a previously unrecognized signaling axis involving ZFAS1-NOP58-SNORD12C/78-EIF4A3/LAMC2 that functions in CRC tumorigenesis and our findings shed new light on our understanding of lncRNAs-snoRNPs-mediated rRNA 2′-O-methylations in CRC tumorigenesis and development.

## Methods

Additional experimental details are included in Additional file 2.

### Collection of the tissue specimen

In this study, human tissue samples were obtained from 157 patients with colorectal cancer, who underwent surgical treatment at the Department of General Surgery of the First Hospital of China Medical University, Department of Medical Oncology of Cancer Hospital of China Medical University between September 2014 and September 2015. This study was approved by the Medical Ethics Committee of China Medical University. All enrolled patients signed the written informed consent form according to the relevant regulations. The tissues of CRC and matched adjacent-tumor controls were snap frozen immediately in liquid nitrogen after separated and stored at − 80 °C before using. The inclusion, exclusion criteria, as well as clinicopathological data collection and follow up of the included patients were described in the Additional file 2.

### LncRNAs-snoRNAs microarray assay

GeneChip® Human Transcriptome Array 2.0 (HTA2.0, Affymetrix, USA) was selected and the microarray hybridization, data acquisition were explored by Shanghai OE Biotech Technology Co, Ltd. (Shanghai, China). The raw data have been deposited in Gene Expression Omnibus under an accession number GSE137511. This HAT2.0 designed array contains more than 6.0 million distinct probes covering coding and non-coding transcripts, and covered more than 285,000 full-length transcripts, more than 245,000 coding transcripts, 40,000 non-coding transcripts and 339,000 probes covering exon-exon junctions of the human genomes. These databases such as *Ensembl*, *UCSC*, *NONCODE*, *RefSeq*, *lncRNAdb*, *Vertebrate Genome Annotation* (Vega), *Mammalian Gene Collection* (MGC), *Human Body Map lincRNAs*, as well as related literatures were used to annotate the determined transcripts. The data were analyzed with Robust Multichip Analysis (RMA) algorithm using Affymetrix default analysis settings and global scaling as normalization method, detail shown in Additional file 2.

### Gene expression analysis

Genesrping software (version 13.1; Agilent Technologies) was employed to perform the raw data analysis. Deferentially expressed genes were then identified through fold change as well as *P* value calculated with *t*-test. The threshold of up- and down-regulated genes was set at fold change ≥2.0 and *P* value ≤0.05. Afterwards, gene ontology (GO) enrichment analysis and Kyoto Encyclopedia of Genes and Genomes (KEGG) analysis were applied to determine the roles of these deferentially expressed mRNAs played in these GO terms or pathways. Finally, Hierarchical Clustering was performed to display the distinguishable genes’ expression pattern among the included 6 samples.

### Cell lines and cell culture

All of the human normal intestinal epithelial cell line HIEC and CRC cells including HCT116, SW480, SW620, and HT29 were obtained from Peking Union Medical College Cell Resource Center (PUMCCRC, Beijing, China). The cells were cultured and maintained under standard cell media and conditions. Specifically, HCT116 were maintained in RPMI-1640 (BI, Israel), SW480 and SW620 cells were grown in L15 (HyClone, USA), HT29 were in McCoy’s 5A (BOSTER Biotech, China), and HIEC were cultured in Dulbecco’s modified Eagle’s medium (DMEM, Invitrogen, USA) plus 10% (*v*/*v*) fetal bovine serum (FBS), and 1% penicillin-streptomycin (Invitrogen, USA). HEK293T cells (from PUMCCRC) were maintained in DMEM plus 10% FBS. All of these cells were grown at 37 °C with a 5% CO_2_ cell culture incubator. In this study, all of the cells used were genotyped by STR analysis and determined routinely for *Mycoplasma* contamination.

### Cell transfection

The plasmid extraction kit was purchased from Sangon Biotech (Shanghai, China). All of the *shRNA* and overexpressing ZFAS1, NOP58 plasmids were described in Additional file 2, and the plasmids nucleotide *shRNA* sequences were listed in Table S1 and Table S2. Cells were plated on 6-well plates to 60–70% confluence and transfected with 1μg/ml Lipofectamine 3000 (Invitrogen, Carlsbad, CA, USA) according to the manufacturer’s instructions.

### Reverse transcription-PCR (RT-PCR) assays and qPCR assays

According to the manufacturer’s instructions (see detail in Additional file 2). Total RNA was extracted from tissues or cells by using TRIzol reagent (Invitrogen, USA). For the RT-PCR assay, the reverse transcription was performed from RNA to cDNA and PCR analyses were performed by a PrimeScript™ RT-PCR Kit (Takara, Japan). Quantitative real-time PCR (qPCR) was determined by SYBR Green I mix (Toyobo, Japan) in triplicate based on an Applied Biosystems 7500HT Real-Time PCR System. The mRNA relative expression was normalized to reference genes GAPDH and/or U6. The reaction assays and primers used for qPCR were listed in Table S3 and Table S4.

### Cell proliferation assays

Transfected HCT116 and SW620 cells were seeded in 96-well plates (100 μl/well) at the density of 5 × 10^3^ cells/well for 24 h. Cell viability was determined for 24, 48, 72 and 96 h by a Cell Counting Kit-8 (CCK8, Bestbio, China) according to manufacturer’s instructions. The absorbance of each well were measured and obtained the OD values at 490 nm with a microtiter plate reader (BioTek, USA). Each time point was assayed in triplicate, and the experiment was replicated 3 times.

### Flow cytometry assays

Cells were harvested and washed twice with cold 1 × PBS. For cell cycle arrest analysis, cells were fixed with 70% ethanol and stored at 4 °C overnight. After re-hydration with PBS, cells were treated with 20 μl of RNase A (2 μg/ml), and incubated at 37 °C for 30 min. Cells were then stained with propidium iodide (PI, 50 μg/ml) for 1 h at 4 °C. For cell apoptosis analysis, cells were re-suspended with 100 μL of 1× Annexin V binding buffer, and incubated with 5 μL of Annexin V-PE for 15 min and 5 μL of 7-AAD for 5 min in a darkroom at room temperature. Finally, cells were analyzed by FACScalibur flow cytometer (BD, USA).

### Western blot analysis

Cells were harvested and lysed by 1 × SDS buffer. Lysates were sonicated and centrifuged (13,000 rpm, 4 °C) for 10 min. Proteins were separated by 8–12% SDS-PAGE and transferred to polyvinylidene fluoride membranes (Millipore, Bedford, MA). Membranes were immunoblotted with anti-rabbit NOP58 (1:1000), EIF4A3 (1:1000) (Proteintech, Chicago, USA; Abcam, UK) and anti-mouse LAMC2 (1:500), GAPDH (1:2000) (Abcam, UK; Zsbio, Beijing, China), and then were incubated with hybrid secondary antibody, and the data was collected by FluorChem V2.0 (Alpha Innotech Corp, USA).

### Immunofluorescence

Cells were grown on cover slides, fixed, and stained with indicated antibodies. Antibodies used for immunofluorescence were as follows: NOP58 (bs-19318R, 1:100, Bioss, Beijing, China), Alexa Fluor anti-rabbit IgG (#4412, 1:500, Cell Signaling Technology). Cell nuclei were counterstained with DAPI (Beyotime, Shanghai, China). Image acquisition was performed on a confocal laser scanning microscope under a 40 × objective (Nikon, Japan).

### Co-localization of LncRNA/snoRNA and protein expression

Cells were cultured on cover slides and fixed normally following the steps of immunofluorescence. Then RNA in situ hybridization was also performed following the kit instructions above except counterstaining with 0.1% Hematoxylin. Next, the cell was continued to stain with indicated NOP58 antibody, Alexa Fluor anti-rabbit IgG and DAPI as immunofluorescence. Similarly, Nikon C2 plus confocal microscope were used to obtain images under a 40 × objective (Nikon, Japan).

### Tissue microarray (TMA) and immunohistochemistry (IHC)

TMA and IHC method were performed as previously described with brief modification [32]. Briefly, the sections (4 μm) were deparaffinized with xylene, rehydrated in a graded alcohol series, and washed in distilled water. Then, sections were incubated in primary antibody of NOP58 (bs-19318R, 1:100, Bioss, Beijing, China) overnight at 4 °C, followed by incubation with biotinylated secondary antibodies for 30 min at 37 °C. The slides were incubated with horseradish peroxidase coupled streptavidin for an additional 30 min (LSAB kit; Dako, Glostrup, Denmark), and stained with DAB (3, 3-diaminobenzidine). Sections were counterstained with hematoxylin, dehydrated, and mounted. Protein expression levels were observed and counted under a microscope (Eclipse 8i, Nikon, Japan), and the evaluation analysis was described in Additional file 2.

### RNA in situ hybridization (ISH) assay

In situ hybridization was performed strictly following the kit instructions (Boster, Wuhan, China). Before prehybridized in prehybridization solution at 42 °C for 2 h, slides were deparaffinized and deproteinated, then incubated with a digoxin-labeled probe solution (Dilute 4 times with 1 × PBS) at 37 °C over night (Specific probe sequences were shown in Table S5). After stringent washing, the slides were exposed to a streptavidin- peroxidase reaction system and stained with DAB (Zsbio, Beijing, China) for 2 min. Then 0.1% Hematoxylin (Solarbio, Beijing, China) was used to counterstain the slides for 5 min. ZFAS1 expression levels were observed and counted under a microscope (Nikon, Tokyo, Japan), and the evaluation analysis was described in Addition file 2.

### RNA stability assay

SW620 cells were transfected with *shZFAS1#1*, *ASO-SNORD12C* followed with a treatment by actinomycin D (ActD, CAS#:A4262, Sigma) at a final concentration of 5 μg/mL for 0.5, 1, 2, 3, and 6 h. Total RNA was extracted and analyzed by qRT-PCR. Then, the calculation of RNA turnover rate and half-life (*t*_1/2_) of SNORD12C, SNORD78, NOP58, EIF4A3, and LAMC2 were determined according to the previous publications [33]. Since ActD treatment results in transcription stalling, the change of RNA concentration at a given time (*dC/dt*) is proportional to the constant of RNA decay (*K*_decay_) and RNA concentration (*C*) as shown in the following equation:

$$ dC/ dt=-{k}_{decay}C $$

Thus the RNA degradation rate *k*_*decay*_ was estimated by:

$$ \mathit{\ln}\left(C/{C}_0\right)=-{k}_{decay}t $$

When 50% of RNA is decayed (i.e., *C/C*_*0*_ = 1/2), the equation below can be used to calculate the RNA half-life (*t*_1/2_):

$$ \mathit{\ln}\left(1/2\right)=-{k}_{decay}{t}_{1/2} $$

From where:

$$ {t}_{1/2}=\mathit{\ln}2/{k}_{decay} $$

### Translation inhibition assay

Briefly, the SW620 cells were cultured for one dish at each time point and then transfected with ASO-Sramble, ASO-SNORD12C and ASO-SNORD78. After 48 h, the cells were treated with translation inhibitor, cycloheximide (CHX, CAS#:C7698, Sigma) with a concentration of 200 μg/ml in the fresh cell medium. Thereafter, the cells were incubated with CHX based on the different time points (0, 1, 2, 3, 4, 6 and 9 h). The zero hour represents the start time of treatment with CHX. The total protein was isolated according to the time courses. Finally, the expression levels of LAMC2, EIF4A3 were measured with GAPDH as the internal control assayed by western blot method.

### RNA pull-down assay

Briefly, biotin-labelled ZFAS1 oligonucleotide (probe sequence shown in Table S6) were conjugated to Streptavidin agarose resin beads. Then, the ZFAS1-conjugated streptavidin beads were then incubated with nuclear extract in binding buffer at 4 °C overnight. After washing with 1 × binding buffers, RNA-protein complexes were dissolved in 1× SDS buffer, and analyzed by western blot assay (see Additional file 2 for details).

### RTL-P assay for rRNA 2′-O-methylation

For the detection of 2′-O-methylation of SNORD12C and SNORD78, RT-PCR was conducted referenced the previous publications with some modifications [34], RT was conducted in 25 μl reaction cocktails with 100 ng of total RNA, 50 μM specific RT primers were denatured at 70 °C for 10 min, and then placed on ice, shown in Table S7 and Table S8. Next, the RT buffer, 200 U M-MLV reverse transcriptase (Takara), 40 U RNasin Ribonuclease inhibitor (Takara) and a low (10 μM) or high (1 mM) concentration of dNTPs were mixed with an initial annealing step at 42 °C for 1 h and then heated at 70 °C for 15 min. Then the PCR reaction was determined and the PCR products were separated on 2% agarose gels, and visualized by UV-trans-illumination.

### Double-stranded primer based on single-stranded toehold (DPBST) assay

Total RNA was extracted from cells by using TRIzol reagent (Invitrogen, USA). The condition of RNA reverse transcription to cDNA was modified by adding low or high dNTPs in a Reverse Transcription Kit (Takara, Japan). The specific primer was named as double-stranded primer based on single-stranded toehold. The BST dsPrimers and reaction assays of SNORD12C and SNORD78 were listed in Table S9 and Table S10. Finally, qPCR assay was determined and the CT curves was obtained using TB Green premix Ex TaqII (Takara, Japan) in triplicate.

### Xenograft mice experiment

All protocols used followed the Regulations of Experimental Animal Administration issued by the Ministry of Science and Technology of the People’s Republic of China. The 4-week-old BALB/c-nu mice were purchased from Shanghai Laboratory Animal Center (Shanghai, China). Before the experiments, the mice were acclimatized to the new environment for one week. 5 × 10^6^ HCT116 cells were subcutaneously injected into the right armpit region. When the tumors were visible, the mice were randomly divided into four groups. The weight and tumor size of the mice were measured every 5 days. Simultaneously, the survival of mice was tracked and recorded. About 5 weeks after injection, half of the mice in each group were sacrificed and the subcutaneous tumors were isolated and measured, the rest was observed for survival until the sixtieth day. Also, the tumor tissues were fixed in 10% formalin for further research.

### Statistical analysis

All of the statistical analysis was employed using SPSS 19.0 software package (SPSS Inc. Chicago, USA), and GraphPad Prism 7.0 software (GraphPad, USA). The data are presented as mean ± standard deviation (*s.d.*) or median (quartile). Student’s *t*-test or *Wilcoxon T-*test was performed to analyze the significant differences of the paired and unpaired continuous variables. *Pearson χ*^*2*^or Fisher’s exact test was conducted to analyze the expression or distribution differences of the variables. Kaplan-Meier method, Log-rank test, and univariate Cox proportional hazard regression analysis were used to estimate the potential prognosis associated indicators. *P-*values were two sides, and *P* < 0.05 was considered statistically significant in all tests.

## Results

### Dysregulated lncRNAs, snoRNAs, and their co-expressing genes

**Fig. 1:**
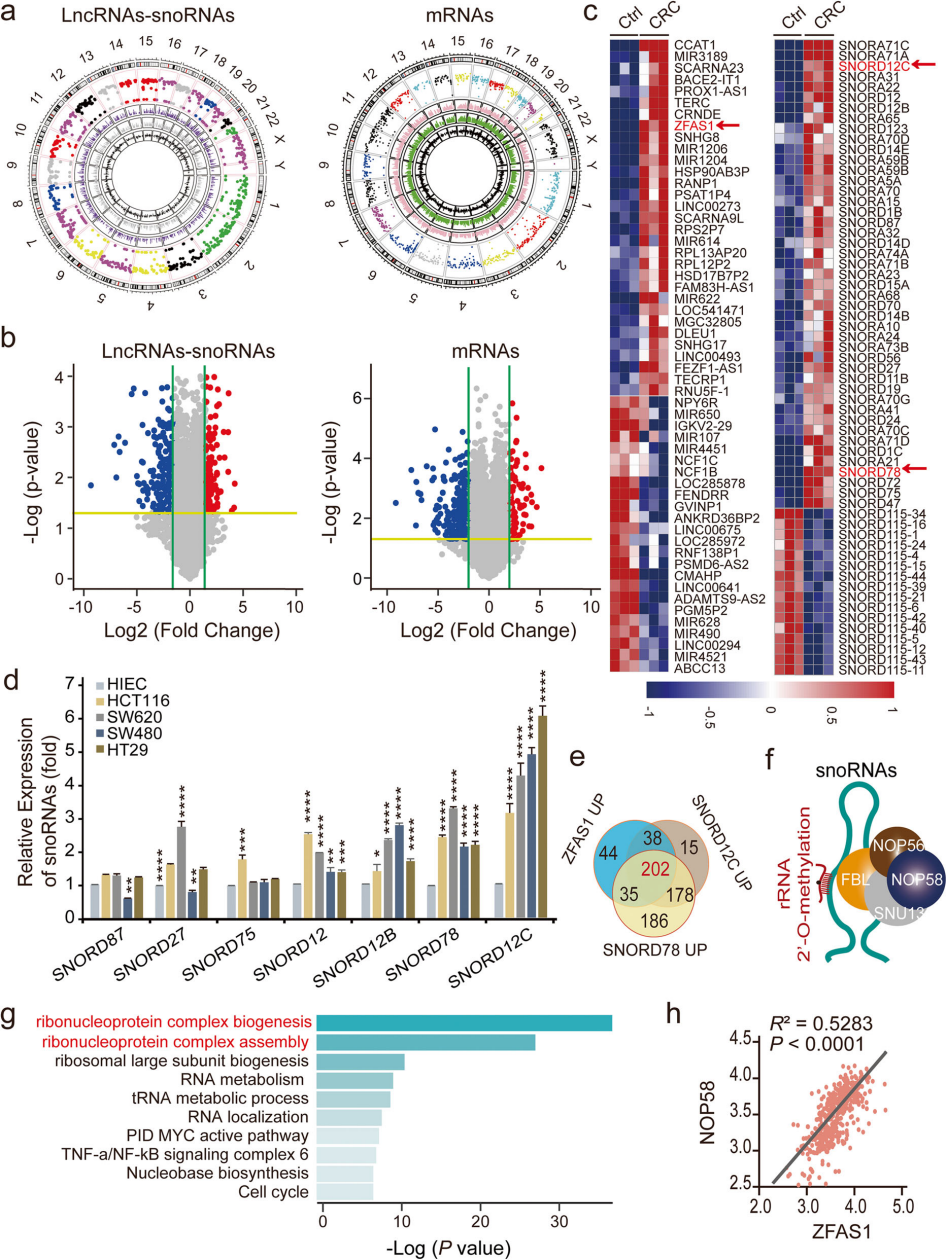
The correlation between ZFAS1, SNORD12C and SNORD78 expression in CRC paired tissues and TCGA. **a**, A circular diagrams from the most inner circle to the most outer circle represent the log2 fold change value of down-regulated or up-regulated differentially expressed LncRNAs, snoRNAs, and mRNAs of the CRC compared with matched adjacent-tumor control tissues (*n* = 3, *P* < 0.05), the gene expression value of matched adjacent-tumor control tissues, the gene expression value of CRC, the different chromosome location of the genes in different colors, and the ruler of chromosome. **b**, The volcano plot of LncRNAs, snoRNAs, and mRNAs expression among included 3 pairs of CRC tissues and adjacent-tumor control tissues (*n* = 3, Log 2 FC = 2.0, *P* < 0.05). **c**, Hierarchical cluster heat map illustrating the most differentially expressed LncRNAs and snoRNAs in CRC and corresponding paired adjacent-tumor control tissues, selected top 30 up-regulated or down-regulated genes (*n* = 3, *P* < 0.05). Red in heat map denotes upregulation. Blue denotes downregulation. **d**, The expression levels of screened snoRNAs in normal intestinal epithelial HIEC cell and CRC cells including HCT116, SW620, SW480, and HT29 detected by qRT-PCR assays. Values are the mean ± s.d. of *n* = 3 independent experiments. **e**, The Venn plot showing the co-expression genes of ZFAS1, SNORD12C and SNORD78 in CRC microarray enrichments (*n* = 3) in our CRC cohort. **f**, The schematic diagram of 2′-O-methylation of ribosomal RNA catalyzed by C/D box snoRNP complexes. **g**, GO pathways enrichment analysis of the up-regulated co-expression genes of ZFAS1, SNORD12C and SNORD78, and the related biological functions. **h**, The linear correlation analysis representing the relation of NOP58 expression levels with ZFAS1 upon TCGA CRC database. *, *P* < 0.05; **, *P* < 0.01; ***, *P* < 0.001; ****, *P* < 0.0001

To explore the dysregulated lncRNAs-snoRNAs-mRNAs and their correlations and co-expression enrichment network, we performed differential expression profiling analyses based on Affymetrix GeneChip microarray that included three CRC patient tissues samples and their matched tumor-adjacent normal tissues (*n* = 3). Significant differences between these two groups were indicated by a ≥ 2-fold change and *P-*value < 0.05, as illustrated in Fig. 1 a, b. The expression differences for ncRNA (lncRNAs, snoRNAs) and mRNA were distributed widely across all chromosomes, including the sex chromosomes (X and Y) (Fig. 1a). In total, we identified 739 dysregulated ncRNAs, including 654 lncRNAs and 85 snoRNAs, and 1164 dysregulated mRNA in this cohort (Fig. 1a). Specifically, 293 up- and 361 down-regulated lncRNAs, 62 up- and 23 down-regulated snoRNAs, and 469 up- and 695 down-regulated mRNAs were identified in this study that examined in CRC tissues and matched tumor-adjacent tissues (Fig. 1b). The heatmap clustering analysis of the top 30 dysregulated lncRNA expression profiles revealed that ZFAS1 is dramatically upregulated (Log_2_ Fold Change/FC = 6.65), and the snoRNAs such as SNORD12C and SNORD78 was remarkably up-regulated, where the Log_2_ FC values were 5.71 and 6.86, respectively (Fig. 1c, Table S11-S12). Additionally, a dramatically higher expression level of ZFAS1 was observed in the vast majority of cancers, particularly in CRC (*http://www.cbioportal.org/*), based on the TCGA data (Fig. S1a). Furthermore, the Top 9 of up-regulated snoRNAs were selected as candidate indicators including SNORD87, SNORD27, SNORD47, SNORD72, SNORD75, SNORD78, SNORD12, SNORD12B, and SNORD12C based on our microarray analysis (*n* = 3). Thereafter, the expression levels of those indicators were identified in four CRC cell lines including HCT16, SW620, SW480, HT29 and normal intestinal epithelial HIEC cells detected by qPCR assay. Of interest, the expression levels of SNORD12C and SNORD78 dramatically elevated compared with other candidate snoRNAs in these included CRC cells, illustrated in Fig. 1d, and Fig. S1d.

To further investigate the potential functions of ZFAS1 expression and its related snoRNAs in CRC patients, we conducted the intersection of the potential target mRNAs, which were enriched from the up-regulated co-expressed with ZFAS1 and up-regulated co-expressed with SNORD12C and SNORD78 by the Bioinformatics & Evolutionary Genomics platform (http://bioinformatics.psb.ugent.be/webtools/Venn/). Subsequently, 202 potential target genes were intersected among these interaction networks, and these networks mainly consisted of components of the C/D box small nucleolar ribonucleoproteins (snoRNPs) such as NOP58 (Fig. 1e, f, Table S14). Based on the TCGA dataset (*n* = 638), NOP58 expression levels were also significantly increased in the CRC tissue samples compared with those in the healthy donors (*n* = 51), which was in contrast to levels of other snoRNPs such as NOP56, SNU13, and FBL (Fig. S1b). Importantly, heat map clusters revealed that NOP58 was dramatically up-regulated in the included CRC tissues and the matched tumor-adjacent normal tissues (Fig. S1c, Table S13). As expected, the Top 2 results of the Gene Ontology (GO) analysis indicated that the potential functions were focused primarily on the regulation of ribonucleoprotein complex biogenesis and ribonucleoprotein complex assembly (Fig. 1g). Further correlation analyses verified that NOP58 showed stronger associated with the expression of ZFAS1 than those indicators such as NOP56, SNU13, and FBL (Fig. 1h, Fig. S1e, Table S14).

### Correlations among the levels of ZFAS1, SNORD12C, SNORD78, and NOP58 in CRC cells

**Fig. 2:**
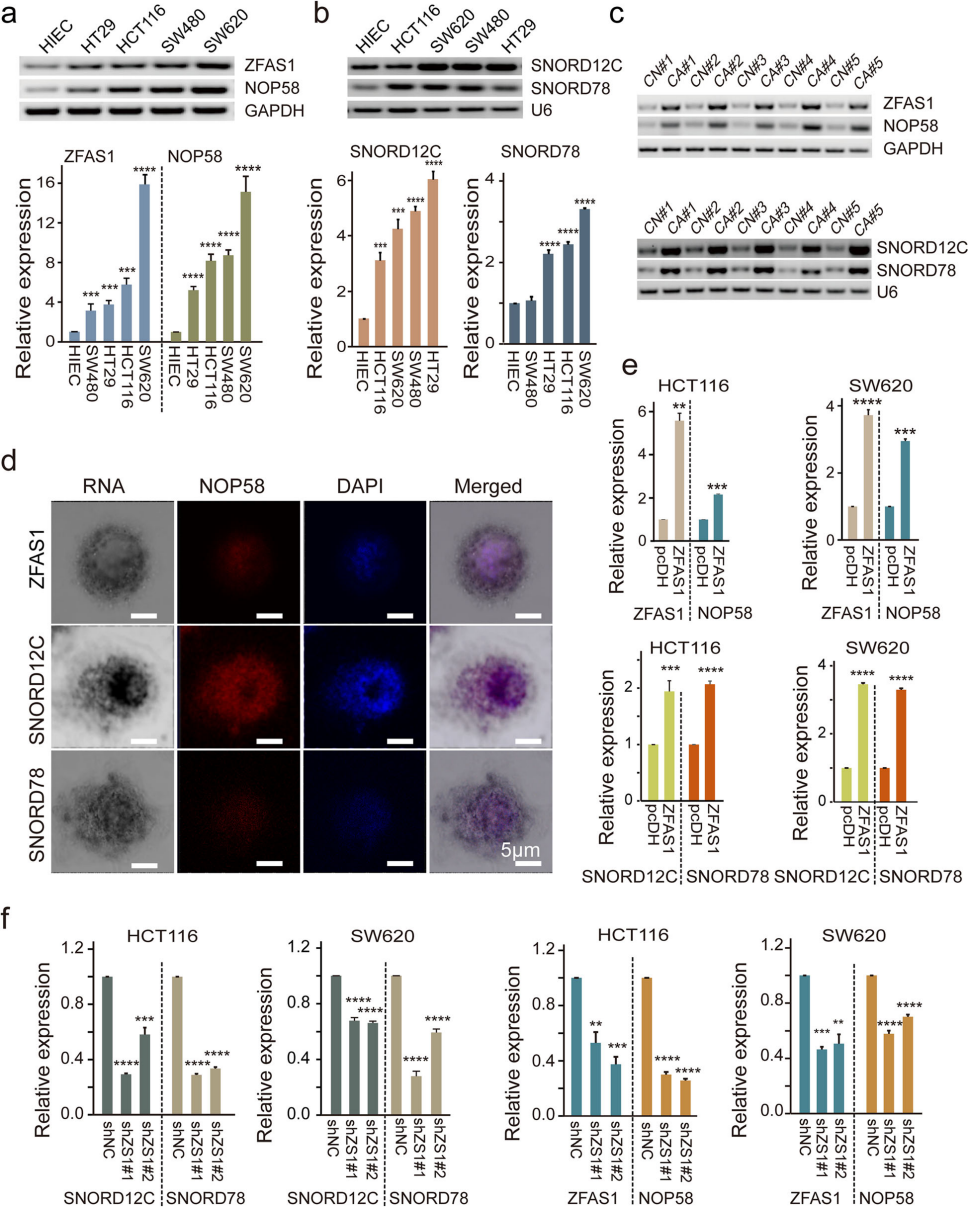
The expression of ZFAS1, SNORD12C, and SNORD78 and subcellular localization. **a** and **b**, Expression levels of ZFAS1, NOP58 (**a**) and SNORD12C or SNORD78 (**b**) in CRC cell lines including HT29, HCT116, SW480, and SW620 and normal intestinal epithelial cell line HIEC detected by RT-PCR and qPCR assay. Values are the mean ± s.d. of *n* = 3 independent experiments. **c**, Representative images of ZFAS1, NOP58, SNORD12C and SNORD78 expression in paired CRC and matched adjacent normal tissues determined by RT-PCR assays (5 representative data was shown). **d**, Co-localization of ZFAS1, SNORD12C and SNORD78 with NOP58 protein detected by ISH and IF assays in HCT116 cells. Scale bar = 5 μm. **e** and **f**, The expression of ZFAS1, NOP58, SNORD12C, and SNORD78 after overexpression or silencing ZFAS1 in both of HCT116 and SW620 cells by qRT-PCR assay.*, *P* < 0.05; **, *P* < 0.01; ***, *P* < 0.001; ****, *P* < 0.0001

To further demonstrate the potential influence of ZFAS1 expression on *SNORD12C*, *SNORD78*, and NOP58, we detected the expression levels of these genes in 4 CRC cells (HT29, HCT116, SW480, and SW620) and in normal control HIEC cells. Our results revealed that ZFAS1 was expressed at a high level, and this expression was accompanied by elevated expression of SNORD12C, SNORD78, and NOP58 in CRC cells as indicated by both RT-PCR and qPCR assays (Fig. 2a, b). Similar results were obtained from 30 pairs of CRC tissues and matched tumor-adjacent normal tissues, where an elevated expression of ZFAS1 in CRC tissues and a further consistently up-regulated of SNORD12C, SNORD78, and NOP58 were detected by RT-PCR and qPCR assays (Fig. 2c, Fig. S2). Importantly, the linear regression analysis further identified the positive correlation among those indicators in our included CRC tissues and controls, shown in Fig. S3. These findings suggested that ZFAS1 expression was positive correlated with the expression of SNORD12C, SNORD78, and NOP58 in CRC cells and tissues. More importantly, co-localization assays using ISH combined with IF revealed that the majority of ZFAS1, SNORD12C, and SNORD78 were co-distributed and co-located with NOP58 within the cell nucleus of the HCT116 cells (Fig. 2d). Additionally, the ectopic ZFAS1 expression caused a substantial elevation in NOP58 and SNORD12C/78 expression levels in HCT116 and SW620 CRC cells (Fig. 2e). In contrast, ZFAS1 knockdown significantly inhibited the expression levels of NOP58 and SNORD12C/78 in CRC cells (Fig. 2f).

Taken together, these data indicated that ZFAS1 expression was up-regulated companied by its correlated SNORD12C/78 and NOP58 in human CRC cells and tissues. A positive regulation pattern was further identified between ZFAS1 and SNORD12C/78 and NOP58 in paired CRC tissues and by interfering ZFAS1 expression in CRC cells.

### Effect of ZFAS1 on RNA stability and rRNA 2′-O-methylation

**Fig. 3:**
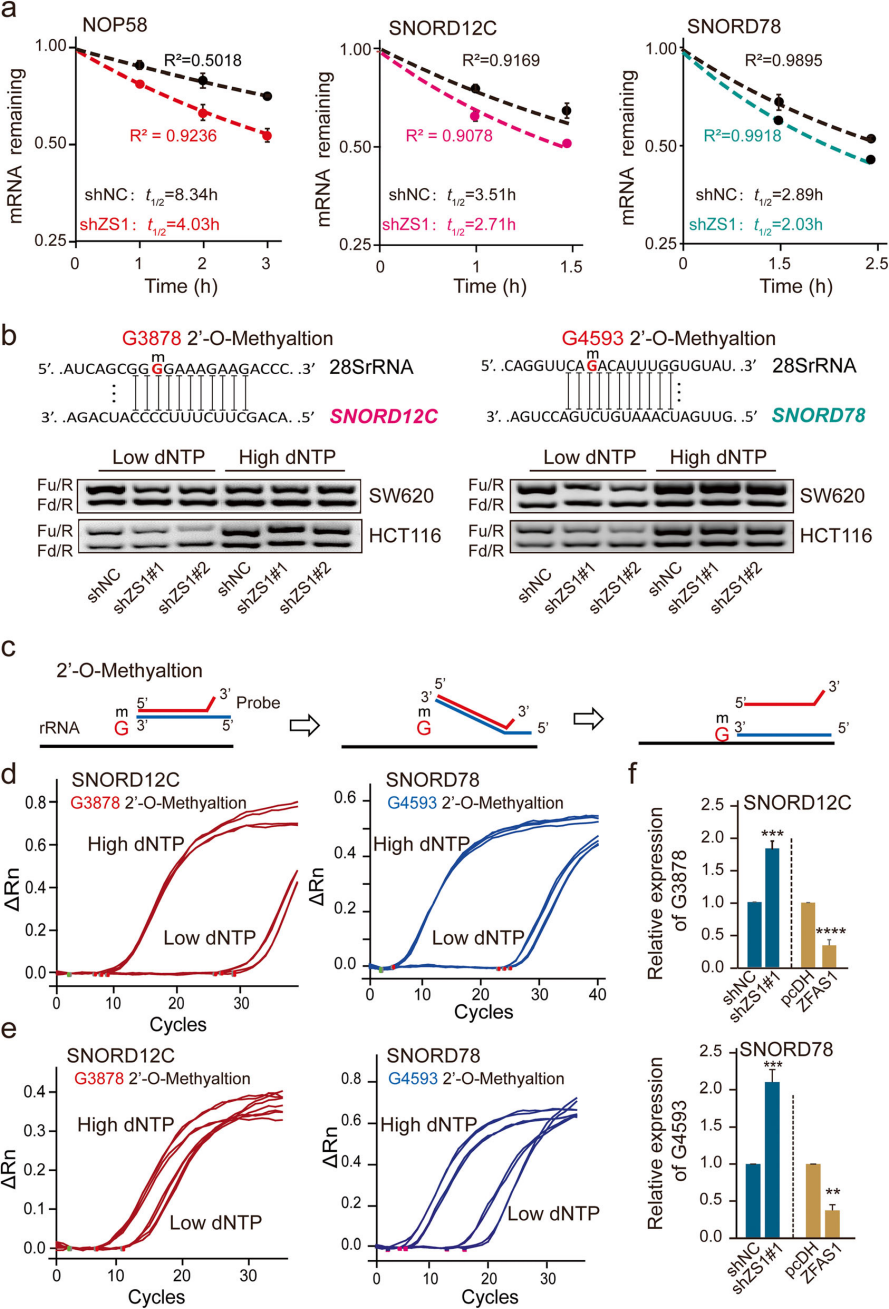
*ZFAS1 inhibits RNA stability of SNORD12C, SNROD78, and the specific sites of rRNA 2′-O-methylation*. **a**, Reducing NOP58, SNORD12C, and SNROD78 half-life (*t*_*1/2*_) by silencing ZFAS1 in SW620 cells. Values are the mean ± s.d. of *n* = 3 independent experiments. **b**, The rRNAs 2′-O-Me activities of G3878 site and G4593 site were decreased after ZFAS1 knockdown in SW620 and HCT116 detected by RTL-P assay. **c**, The schematic structures showing a novel method called double-stranded primer based on single-stranded toehold (DPBST) assay for detecting rRNAs 2′-O-Me levels. **d** and **e**, The 28S rRNA G3878 and G4593 sites of 2′-O-Me mediated by SNORD12C or SNORD78 at the high or low dNTPs conditions after silencing ZFAS1 (Upper) or overexpression ZFAS1 (Lower) in SW620 cells detected by DPBST assays. **f**, The DPBST detecting statistical results of 2′-O-Me by qPCR assay. *, *P* < 0.05; **, *P* < 0.01; ***, *P* < 0.001; ****, *P* < 0.0001

Based on the observation that the knockdown of ZFAS1 inhibits the expression of snoRNAs, SNORD12C/78, and NOP58, we next investigated if the changes in the expression levels of mRNAs and snoRNAs after ZFAS1 silencing were due to accelerated mRNA decay. To achieve this, we conducted the RNA stability assays in SW620 cells with the treatment of ActD. As expected, the half-lives of RNAs remaining significantly decreased in those indicators including NOP58, SNORD12C, and SNORD78 compared with the NC group (Fig. 3a). Furthermore, two methods including RTL-P and DPBST assays were explored to confirm the 28S rRNAs 2′-O-Me activity mediated by SNORD12C or SNORD78. Notably, RTL-P assays demonstrated that ZFAS1 knockdown dramatically reduced the 28S rRNA 2′-O-Me activity at the G3878 site modified by SNORD12C and the G4593 site modified by SNORD78 under the lower dNTPs concentrations, however, no significant difference was observed under the higher dNTPs conditions (Fig. 3b, Fig. S3f, g). Similarly, DPBST assays further revealed that the 2′-O-Me activities modified by SNORD12C or SNORD78 were significantly decreased upon ZFAS1 knockdown, meanwhile, overexpression of ZFAS1 elevated the activities of 2′-O-Me (Fig. 3c-f).

Taken together, our results indicate that ZFAS1 exerts a significant effect on the RNA stability and 2′-O-Me activities mediated by SNORD12C or SNORD78, ultimately affecting CRC tumorigenesis and epigenetic 2′-O-Me levels of target 28S rRNAs.

### Effects of ZFAS1 on CRC cell proliferation and apoptosis

**Fig. 4:**
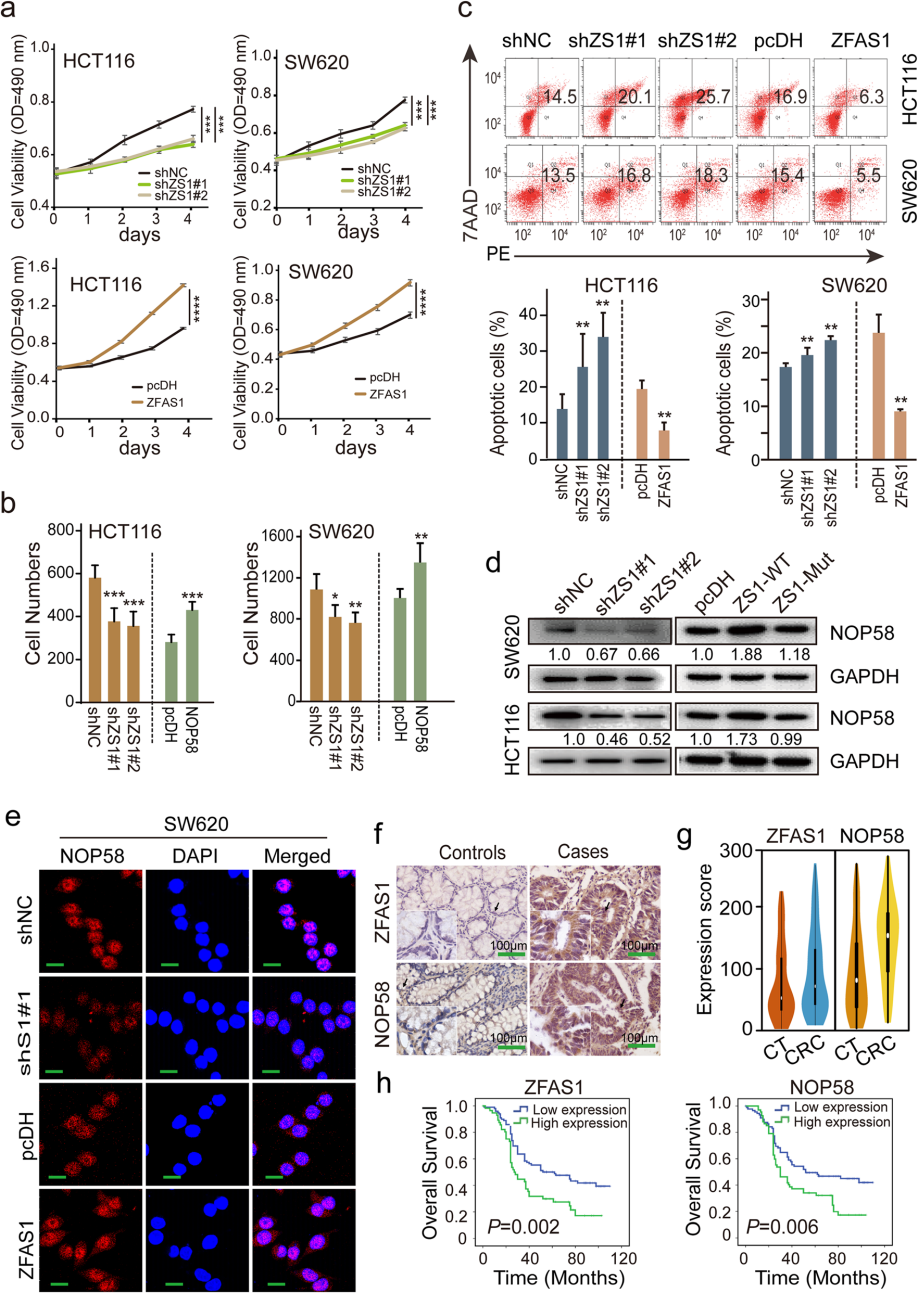
Effects of ZFAS1 on cell proliferation and apoptosis by regulating NOP58 expression in CRC cells. **a**, CCK8 assays were used to identify the cell proliferation abilities upon ZFAS1 silencing or overexpressing in HCT116 and SW620 cells. *n* = 3 independent experiments. **b**, The migrated cell numbers were determined after ectopic or knockdown ZFAS1 in HCT116 and SW620 cells. **c**, The percentage (%) of cell apoptosis was detected upon ZFAS1 overexpressing or silencing in HCT116 and SW620 cells by Flow cytometry. *n* = 3 independent experiments. **d** and **e**, The NOP58 expression was conducted after overexpressing or knocking down ZFAS1 by Western blot (d), and IF assays (e). **f**, ISH and IHC methods were used to determine the cell localization and expression levels of ZFAS1 and NOP58 in CRC tissues and matched adjacent-tumor controls (*n* = 157). Scale bars = 100 μm. **g**, Violin charts displaying the expression scores and levels of ZFAS1 and NOP58 between CRC tissues and matched paired adjacent normal tissues (*n* = 157). Nonparametric tests and Median and 95%CI were shown. **h**, Kaplan-Meier curves representing the impact of ZFAS1 and NOP58 on overall survival in this included CRC cohort.*, *P* < 0.05; **, *P* < 0.01; ***, *P* < 0.001; ****, *P* < 0.0001

To further verify the impact of ZFAS1 on the biological mechanisms of CRC, we used CCK8 assays to determine that overexpression of ZFAS1 significantly enhanced the proliferation of HCT116 and SW620 cells, while knockdown of ZFAS1 suppressed the proliferative ability of these two cell lines (Fig. 4a). Additionally, knockdown of ZFAS1 greatly decreased the migrated cells numbers in both HCT116 and SW620 cells, and in contrast, the ectopic expression of ZFAS1 promoted an increase in the numbers of HCT116 and SW620 cells (Fig. 4b). ZFAS1 knockdown also resulted in a marked increase in cell apoptosis, and overexpression of ZFAS1 substantially inhibited apoptosis in both HCT116 and SW620 cells as determined by flow cytometry (Fig. 4c, Fig. S4a). RT-qPCR analyses indicated that the inhibition of ZFAS1 expression positively affected *NOP58* expression in both HCT116 and SW620 cells, and similarly, *NOP58* protein expression levels were also decreased after ZFAS1 inhibition in HCT116 and SW620 CRC cells as determined by WB and IF assays (Fig. 4d, e, Fig. S4 b, c). Similarly, in vitro rescue experiments revealed that NOP58 overexpression reversed the ZFAS1 inhibition effect on CRC molecular characteristics including cell proliferation ability, cell apoptotic rates in both HCT116 and SW620 cells assayed by CCK8 (Fig. S4d), and flow cytometry analysis (Fig. S4e). We next used tissue microarray (TMA) and immunohistochemistry (IHC) to examine a relatively large number of samples that included 157 pairs of CRC and matched tumor-adjacent normal tissues (Fig. 4f). The cut-off value of ZFAS1 or NOP58 expression was determined based on the ROC curve method (Fig. S5 a, b). Consistent with the results in CRC cells, the expression levels of ZFAS1 and NOP58 were elevated in the CRC tissues compared to the levels detected in the tumor-adjacent normal tissues (Fig. 4g), and a large positive linear correlation pattern was confirmed to exist between ZFAS1 and NOP58 within this cohort (Fig. S5c). As expected, the prognostic analysis revealed that elevated ZFAS1 expression significantly correlated with shortened overall survival (OS, *P* = 0.002) and reduced disease-free survival (DFS, *P* = 0.002) (Fig. 4h, Fig. S5d). Consistently, higher expression of NOP58 was significantly associated with poor OS (*P* = 0.006) and DFS (*P* = 0.006) (Fig. 4h, Fig. S5d). Further multivariate Cox regression analyses also confirmed the prognostic values of ZFAS1 and NOP58 after adjusting for confounders including age and pathological pattern at diagnosis for OS and tumor stage for DFS (Table S15-S16).

Collectively, our results indicated that overexpression of ZFAS1 significantly accelerated the progression of human CRC cells by promoting the expression of NOP58 levels; however, the molecular mechanisms and underlying functions require further investigation.

### NOP58 recruitment was accelerated upon ZFAS1-mediated 2′-O-me activities of SNORD12C/78

**Fig. 5:**
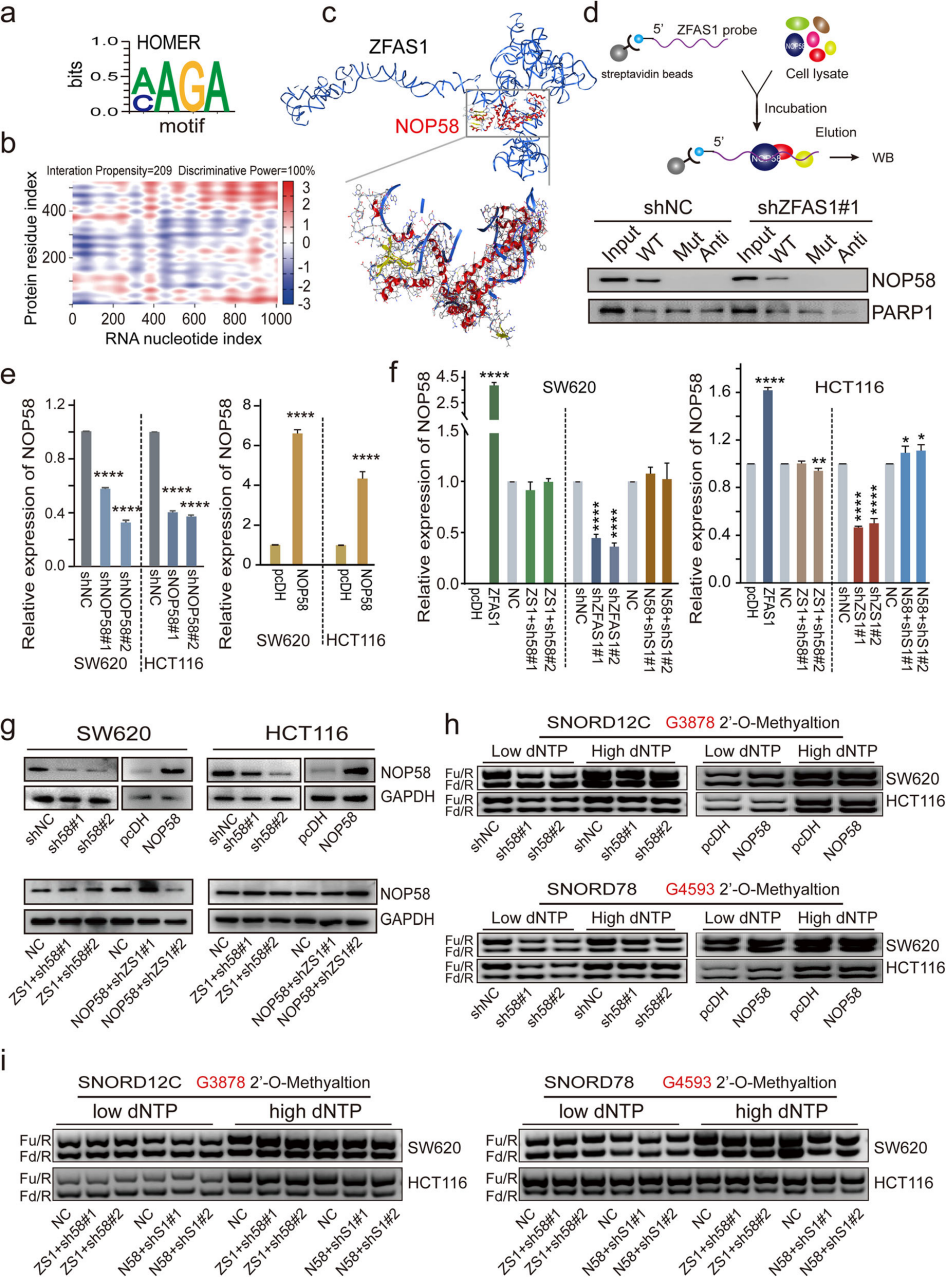
ZFAS1 promotes rRNA G3878 and G4593 2′-O-methylation by targeting NOP58 protein. **a**, CLIP database showing the motif of ZFAS1 targeting NOP58 predicted by online software (http://lulab.life.tsinghua.edu.cn/postar/index.php). **b**, Bioinformatics online software predicting the specific binding sequence and sites of ZFAS1 secondary structure and NOP58 protein (http://www.tartaglialab.com/). **c**, MOE multi-functional docking platform showing the specific docking sites between ZFAS1 tertiary structure and NOP58 protein. **d**, RNA pull-down followed by western blot showed in vitro binding of the ZFAS1-Wild, ZFAS1-Mutant, and antisense RNA probes with NOP58 protein after ZFAS1 silencing in SW620 cells. The biotin labeled probes are presented in Table S6. **e** and **f**, The NOP58 expression after overexpressing or knockdown NOP58 by qRT-PCR assay (**e**), and ZFAS1 rescued NOP58 mRNA expression after co-transfected ZFAS1 and NOP58 (**f**). **g**, Western blot assays detected the NOP58 protein expression after overexpressing, knocking down NOP58, as well as co-transfected ZFAS1 and NOP58 vectors. **h** and **i,** The 28S rRNA G3878 and G4593 2′-O-Me activities after overexpressing or knockdown NOP58 in SW620 and HCT116 cells by RTL-P (h), and ZFAS1 rescued the 2′-O-Me activities after co-transfected ZFAS1 and NOP58 (i).*, *P* < 0.05; **, *P* < 0.01; ***, *P* < 0.001; ****, *P* < 0.0001

To further clarify the molecular mechanism by which ZFAS1 affects the 2′-O-Me activities modified by SNORD12C/78 and its regulation pattern, the catRAPID platform (*http://service.tartaglialab.com*) was employed to evaluate the interaction propensity and discriminative power between the ZFAS1 nucleotide index and the NOP58 residue index (Fig. 5b). As expected, ZFAS1 exhibited significant interaction propensity (value = 209) and discriminative power (100%) for NOP58. The critical binding motif were predicted by *POSTAR2* (*http://lulab.life.tsinghua.edu.cn/postar/*) (Fig. 5a). Furthermore, MOE software was used to identify the direct binding domain and the critical amino acids within NOP58 3D structure, illustrated in Fig. 5c.Thereafter, the biotin-labeled LncRNA-ZFAS1 probes were synthesized containing the binding motif with NOP58 (ZFAS1-WT) or a corresponding mutant sequence (ZFAS1-Mut). RNA pull-down assays further demonstrated that the ZFAS1-WT probe, but not ZFAS1-Mut, significantly pulled down endogenous nuclear NOP58 protein (Fig. 5d), and this enrichment was significantly reduced after silencing ZFAS1 expression, indicating a direct regulation of ZFAS1 with NOP58 by specific binding manner. Next, NOP58 knockdown or overexpression cell models were established by SW620 and HCT116 cells (Fig. 5e). qPCR assays confirmed that NOP58 mRNA expression can be recovered compared with the NC group after co-transfection with *pcDH-ZFAS1* and *shNOP58* or *shZFAS1#1/#2* and *pcDH-NOP58* (Fig. 5f). WB assays further determined that the decreased NOP58 protein expression was retrieved by co-transfection with *pcDH-ZFAS1* and *shNOP58* or *shZFAS1#1/#2* and *pcDH-NOP58* in both SW620 and HCT116 cells (Fig. 5g). More importantly, the 2′-O-Me activities mediated by SNORD12C and SNORD78 were dramatically decreased after knockdown NOP58 expression in both SW620 and HCT116 cells (Fig. 5h, Fig. S6a, b). Similar results from the rescue experiments demonstrated that the 2′-O-Me activities were recovered both in HCT116 and SW620 cells as a result of NOP58 overexpression after co-transfected with shZFAS1#1/#2 (Fig. 5i, Fig. S6c, d).

Taken together, these data suggest that ZFAS1 recruits NOP58 by direct binding that is mediated by 2′-O-methylation activities of SNORD12C and SNORD78, and this recruitment significantly impacts CRC tumorigenesis and development.

### SNORD12C/78-mediated 2′-O-me regulates the translation activity of target genes in a ZFAS1- dependent manner

**Fig. 6:**
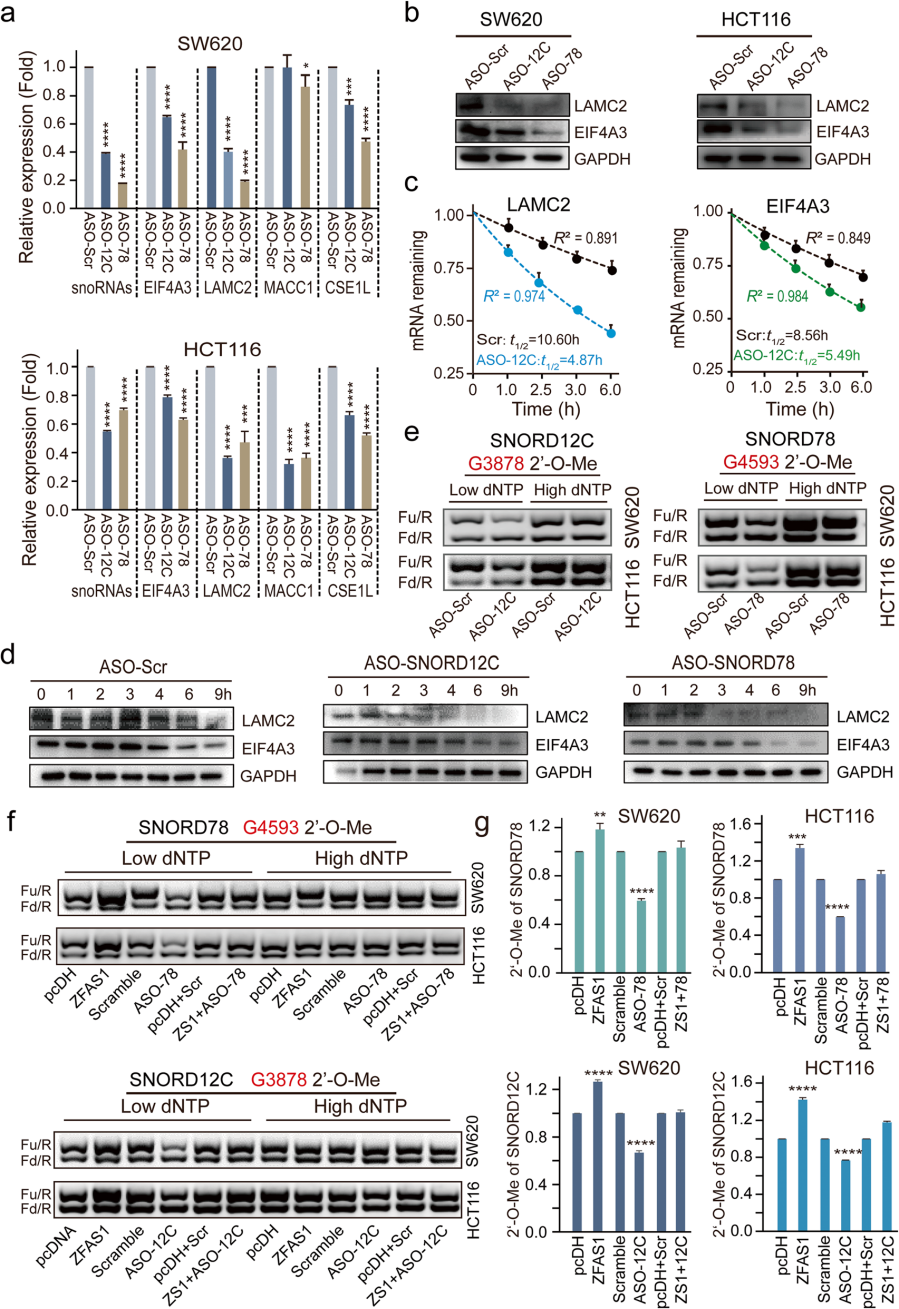
SNORD12C/78-mediated 2′-O-Me modification regulates target genes expression in a ZFAS1-dependent manner. **a** and **b**, EIF4A3 and LAMC2 mRNA expression was determined after knocking down SNORD12C or SNORD78 by qRT-PCR assays(**a**), and Western blot method(**b**). **c**, Reducing EIF4A3 and LAMC2 half-life by silencing SNORD12C treated by the ActD in SW620 cells. **d**, Reducing the translation activity of EIF4A3 and LAMC2 by silencing SNORD12C and SNORD78 treated by the CHX in SW620 cells. **e**, The 28S rRNA G3878 and G4593 2′-O-Me activities were declined after silencing SNORD12C or SNORD78 expression in SW620 and HCT116 cells detected by RTL-P assays. **f**, Overexpressing ZFAS1 rescued the 2′-O-Me activities under the lower dNTPs conditions after silencing SNORD12C and SNORD78. g, The statistical plot of overexpressing ZFAS1 rescued the 2′-O-Me activities after silencing SNORD12C and SNORD78 *, *P* < 0.05; **, *P* < 0.01; ***, *P* < 0.001; ****, *P* < 0.0001

To further clarify the function of *snoRNAs* in the context of ZFAS1-mediated regulation of CRC cell proliferation and the translation of target genes, we employed enrichment of co-expression analyses between SNORD12C/78 and mRNA expression to search the possible downstream target genes, including EIF4A3, LAMC2, MACC1, and CSE1L, responsible for regulating 2′-O-Me activities. We found that the expression levels of EIF4A3 and LAMC2 expression were significantly decreased upon knockdown of SNORD12C/78 expression as assessed by qPCR and WB assays (Fig. 6a, b). Additionally, RNA stability assays further identified that the half-life of LAMC2 and EIF4A3 mRNA was reduced by knockdown SNORD12C expression or by treatment with ActD in SW620 cells compared to the half-life in cells treated with empty vector (negative control) (Fig. 6c). In agreement with these findings, the protein expression levels of LAMC2 and EIF4A3 were measured after treatment with the CHX, and we observed that the expression levels gradually decreased throughout this time course (Fig. 6d) in SW620 cells. Finally, the 2′-O-Me activities modified by SNORD12C or SNORD78 were significantly decreased after silencing SNORD12C or SNORD78 expression in both of SW620 and HCT116 cells (Fig. 6e, Fig. S7a, b). Similarly, this decrease was rescued by ZFAS1 overexpression when co-transfected with ASO-SNORD12C or ASO-SNORD78 assessed by RTL-P assays in both HCT116 and SW620 cells (Fig. 6f, g), suggesting the existence of a 2′-O-Me mediated regulation between ZFAS1 and SNORD12C or SNORD78.

### ZFAS1 inhibits cell proliferation by interacting with NOP58 in vivo

**Fig. 7:**
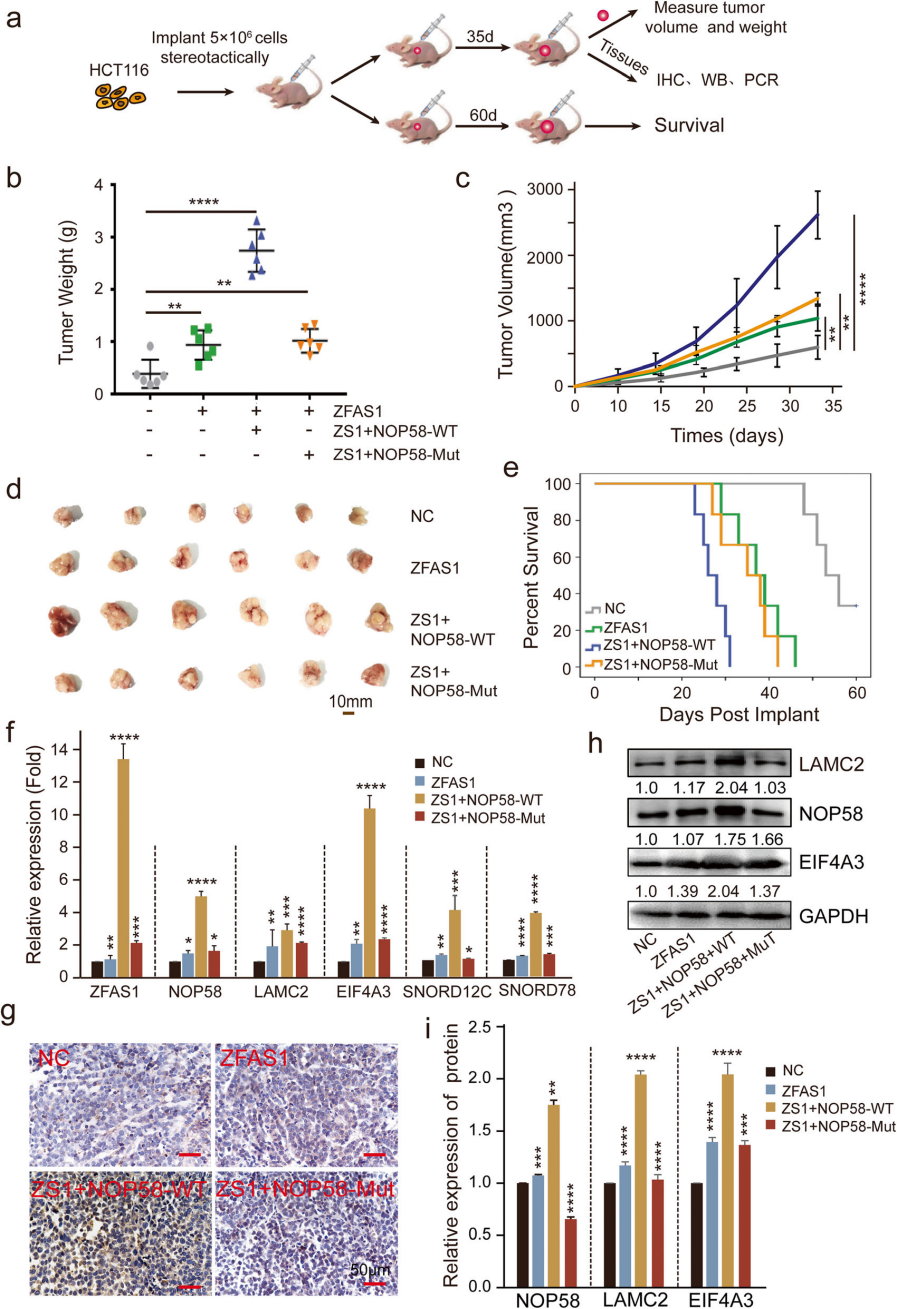
ZFAS1 inhibited proliferation by targeting NOP58 protein in vivo. **a**, Schematic diagram of xenografts in BALB/c nude mice by inoculating HCT116 cells that were stably co-transfected with ZFAS1, ZFAS1-NOP58-Wild, and ZFAS1-NOP58-Mut, as well as the control with empty vector at their right armpits. Then half of the xenografts were sacrificed at the 35th day after injection and the other half were tracked until death. **b**, Mean tumor weight of each group xenografts in nude mice. Data are shown as mean ± s.d., *n* = 6 for each group. **c**, Mean tumor volumes on different days for each group xenografts in nude mice. Data are showed as mean ± s.d., *n* = 6 for each group. **d**, Representative tumors size excised on day 35 are shown. **e**, Kaplan-Meier graph showing overall survival of each group, *n* = 6. **f**, qPCR assays were performed to determine the (m) RNA expression of ZFAS1, NOP58, LAMC2, EIF4A3, SNORD12C, SNORD78 in above each group. **g, h** and **i**, IHC assay and western blot to detect the protein expression of NOP58 in xenografts tumor tissues of each group. The groups were as follows: NC (empty vector); ZFAS1 (pcDH-ZFAS1); ZFAS1 + NOP58-Wild (co-transfected with pcDH-ZFAS1 and pcDH-NOP58-Wild); ZFAS1 + NOP58-Mut (co-transfected with pcDH-ZFAS1 and pcDH-NOP58-Mut).*, *P* < 0.05; **, *P* < 0.01; ***, *P* < 0.001; ****, *P* < 0.0001

To evaluate the molecular mechanism of ZFAS1 interaction with NOP58 in the context of CRC in vivo, we first established xenografts in BALB/c nude mice by inoculating HCT116 cells that were stably co-transfected with ZFAS1 and *NOP58-WT*, *NOP58-Mut* vector, or NC controls within the same mice at their right armpits (Fig. 7a). The mice were sacrificed, and the xenograft tumors were removed at the fifth week after implantation or followed up until death. As expected, a dramatic increase in the xenograft tumor weight and tumor volume was observed after the co-transfection with ZFAS1 and the *NOP58-WT* vector compared to that observed after transfection with NC or the overexpressing ZFAS1 vector (Fig. 7b, c, d), indicating that the promotion of tumorigenic potential in CRC cells is a result of ZFAS1 specifically interacting with NOP58 in xenograft mouse models. To further confirm this, we compared the survival times among different groups, and we observed a substantially reduced survival time in xenograft mice within the ZFAS1 and *NOP58-WT* co-transfection group (Fig. 7e), indicating the interaction between ZFAS1 and NOP58 acts as a synergistic risk factor in the prognosis evaluation of CRCs. Additionally, in agreement with in vitro data, SNORD12C/78, EIF4A3, and LAMC2 expression were significantly up-regulated by the presence of ZFAS1 and *NOP58* in vivo as assessed by qPCR (Fig. 7f), suggesting a direct binding regulated by ZFAS1. IHC and WB analyses further indicated that NOP58 was significantly increased in the group co-transfected with ZFAS1 and the *NOP58-WT* vector, and this was accompanied by an increase in the levels of their targets such as EIF4A3 and LAMC2 (Fig. 7g-i). Collectively, these results demonstrated that ZFAS1 promotes the proliferation and survival of CRCs by targeting NOP58 to modulate its protein translation.

## Discussion

Colorectal cancer (CRC) remains one of the most common digestive system malignancies that exhibit higher morbidity and mortality worldwide [35]. In humans, a complicated set of factors are involved in CRC tumorigenesis, including environmental exposures, inherited genetic mutations (particularly multiple epigenetic modifications), and other factors [36–38]. Despite the use of advanced treatments such as surgical resection, chemotherapy, and/or radiotherapy, the mortality rate of CRC patients has not appreciably improved, and this is likely due to deficiencies in effectively screening for molecular biomarkers. Accumulating evidence indicates that the non-coding RNAs (ncRNAs), specifically long non-coding RNAs (lncRNAs) and small nucleolar RNAs (snoRNAs) of short ncRNAs, can act as master regulators of gene regulation and RNA modification [39, 40]. Defective function of these molecules has become one of the hallmarks of tumorigenesis and cancer development. Thus, insight regarding reliable molecular biomarkers that are involved in the progression and development of CRC is becoming more valuable, as it could considerably facilitate the clinical diagnosis and promote the prognosis of CRC patients.

**Fig. 8:**
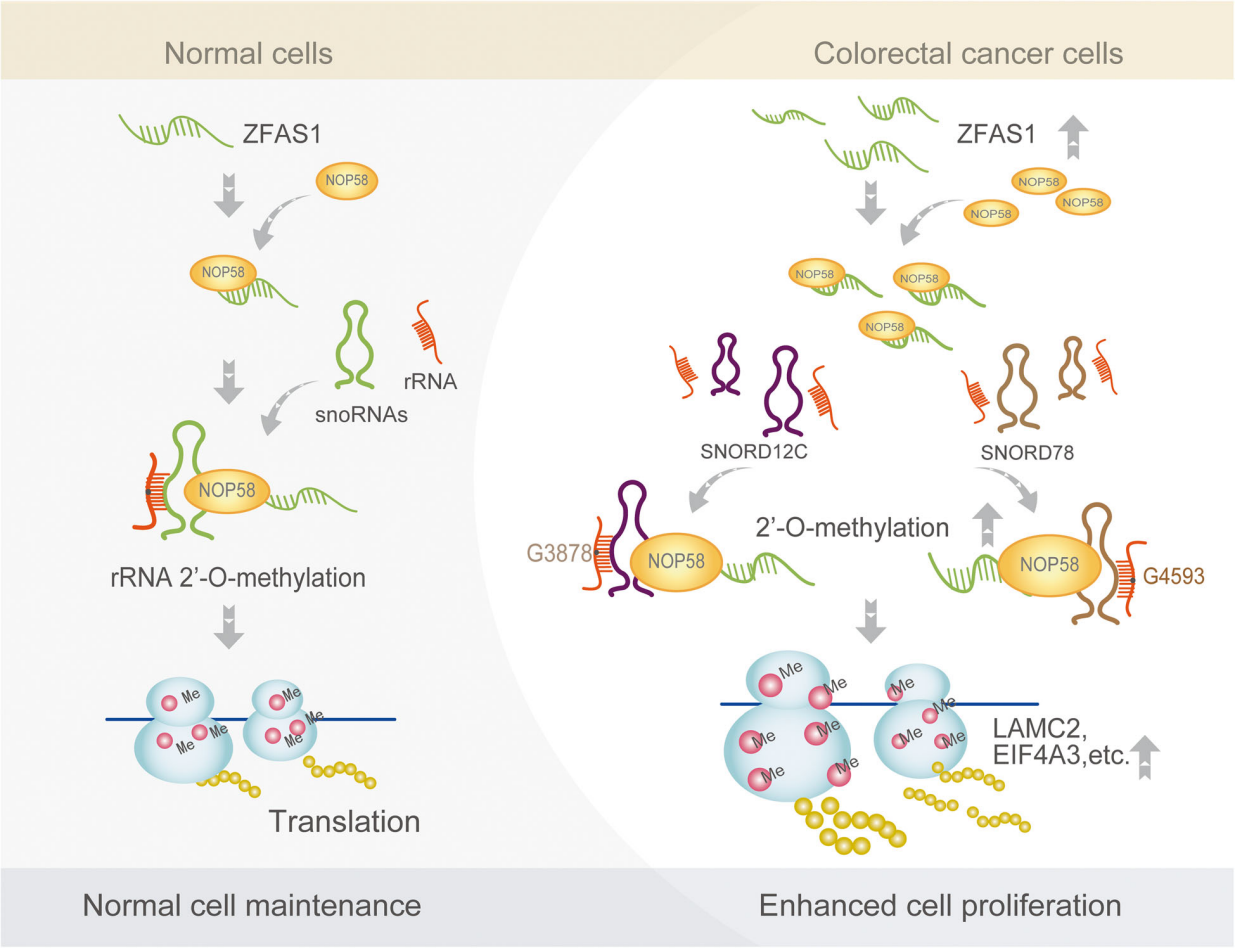
The schematic diagram illustrated that ZFAS1 targeting recruitment NOP58 regulated rRNA 2′-O-methylation mediated protein translation to mediate colorectal cell proliferation. In normal intestinal epithelial cells, ZFAS1, NOP58 and snoRNAs are expressed normally, as well as the level of the rRNA 2′-O-methylation and translation. In CRCs cells and tissues, ZFAS1 is overexpressed, and its binding protein NOP58, which is one of the snoRNPs, is also overexpressed. ZFAS1 increases the snoRNPs formed by the synergistic recruitment of snoRNAs in combination with NOP58, especially SNORD12C and SNORD78.This results in an increase in the level of rRNAs 2′-O-Me modification, which ultimately leads to changes in translational activity and precision of downstream target genes, thereby mediating colorectal cancer proliferation

Ribose 2′-O-Me, the most prevalent internal chemical modification between ribosomal RNA and snoRNAs, plays critical roles in many bioprocesses such as RNA stability and translation fidelity in multiple species ranging from bacteria to mammals [41–43]. Notably, dysregulated levels of C/D box snoRNA correlated with alterations in 2′-O-Me of rRNA, and these alterations can contribute to cancer progression and outcome [44]. The underlying molecular mechanism of the snoRNAs and their related regulation network remain elusive, however, especially in colorectal cancer. Here, our studies identify ZFAS1 and its direct interaction target NOP58 (the core components of snoRNPs) as central to the master C/D box snoRNAs mediated 2′-O-methylation epigenetics modification network that is essential for CRC initiation and maintenance (see the proposed model in Fig. 8). Briefly, in normal cells, ZFAS1 and NOP58 (a direct binding target) are normally expressed, and the C/D box snoRNAs SNORD12C and SNORD78 (specific assembly snoRNP complex) are recruited at normal levels. In CRC cells and tissues, ZFAS1 and its endogenous recruiting protein NOP58 are up-regulated, and this promotes the SNORD12C and SNORD78-mediated 28S rRNAs 2′-O-Me activities at specific sites to substantially increase the translation activation of downstream genes such as EIF4A3, LAMC2, and others. This promotion can be blocked by transfection with a *NOP58*-mutant vector in vivo, and this blockage reverses the expression patterns of the above genes (Fig. 8).

Increasing studies have provided evidence that lncRNAs (e.g., H19, MEG3, MALAT1, etc.) exert their functions in the context of cancer initiation and progression by influencing epigenetic modifications such as DNA CpG methylation and RNA N6-methyladenosine (m6A); however, they have not been observed to influence rRNA 2′-O-methylation [45–47]. In the current study, we demonstrated that ZFAS1 accelerates the recruitment of SNORD12C and SNORD78 through a direct interaction with NOP58, a core component of RNPs, to assemble the corresponding snoRNP complexes. This subsequently promotes the 2′-O-Me of 28S rRNA and ultimately plays an important role in CRC tumorigenesis and clinical outcomes. Recently, critical lncRNAs were found to be dysregulated and to act as oncogenes that influence cell fate decisions by promoting cell proliferation, migration, invasion, and apoptosis inhibition. Similar to the recent studies demonstrating the oncogenic role of ZFAS1 in NSCLC, HCC, gastric cancer, and other cancers [48, 49]. For example, Zhang et al. reported that ZFAS1 involved in gastric cancer initiation and development based on ceRNA network and Wnt/β-catenin signaling axis, which provide new targets and biomarkers for gastric cancer treatment and evaluation [50, 51]. Consistently, our findings demonstrated that ZFAS1 functions as a critical oncogene in CRC and that its expression/function is required for both development and maintenance in CRC cells and tissues. In contrast to the general assumption that noncoding SNORD-host transcripts function only as vehicles to generate snoRNAs, ectopic expression of *ZFAS1* in CRC cells resulted in elevated cell proliferation, invasion promotion, and cell apoptosis inhibition [22]. Additionally, ectopic expression of *ZFAS1* also up-regulated the levels of the SNORD12C (the host gene ZFAS1) and SNORD78, and resulted in elevated NOP58 expression. Interestingly, knockdown of ZFAS1 further resulted in decreased RNA stabilization of NOP58, SNORD12C, and SNORD78 and in reduced levels of 28S rRNA 2′-O-Me at specific sites (SNORD12C: 28S-Gm3878, SNORD78: 28S-Gm4593). Complete distribution profiles of the residues revealed that the biological characteristics of snoRNAs primarily depend upon the types that are associated with a set of core RNPs to assemble stable and functional snoRNP particles to thereby mediate the consequent biological functions. C/D snoRNAs are associated with evolutionarily stable and highly conserved core RNPs, including NOP58, NOP56, SNU13, and with the methyltransferase FBL to guide 2′-O-Me of target rRNAs. Among these proteins, NOP58, as an adaptor in snoRNPs, binds to the conserved C box (RUGAUGA) and D box (CUGA) of C/D box snoRNAs to provide a skeleton and bridge for the entire snoRNP complex, ultimately maintaining the homeostasis of epigenetic modifications [52]. Our current studies further supported that NOP58 can directly interact with ZFAS1 to induce its function, and this complex then participates in C/D box SNORD12C and SNORD78 snoRNP complex assembly that is involved in 2′-O-Me of nucleotides at specific positions in rRNAs that ultimately contribute to CRC tumorigenesis. It is noteworthy that this pattern was confirmed by using the ZFAS1-Mut probe and/or vector with the binding sequence, indicating a direct binding interaction between ZFAS1 and NOP58 that influenced regulation in vitro and in vivo.

2′-O-Me is present within various cellular RNAs and is essential for RNA biogenesis and functionality [34]. Although the importance of rRNA 2′-O-Me was established by suppression of snoRNA expression in a zebrafish model [8], it has not been extensively studied in human cellular translation. Here, we developed a novel method referred to as DPBST (Double-stranded primer based on single-stranded toehold) to detect 2′-O-Me activities in rRNAs. This method allows for precise mapping and superior sensitivity compared to that of previous classical methods such as RTL-P. Using RTL-P and DPBST assays, we further investigated the molecular mechanisms of ZFAS1 in regard to the biological functions of 2′-O-Me, including improvement in RNA stability, fine modulation of its conformation, and its importance in ribosomal translation. Indeed, the 2′-O-Me activities were significantly decreased after treatment with ASO-SNORD12C/78 in CRC cells. In contrast, this decrease was almost completely rescued by ZFAS1 overexpression in cells co-transfected with ASO-SNORD12C/78. Additionally, RNA stability and translation activity of their downstream targets such as EIF4A3 and LAMC2 were significantly affected in this regulatory network. Specifically, knockdown of SNORD12C/78 remarkably decreased the expression, the mRNA remaining half-life, and the translation fidelity of EIF4A3 and LAMC2 in CRC cells. These data indicated that ZFAS1 functioned as an endogenous regulator to increase the expression of SNORD12C/78 by directly binding to NOP58. SNORD12C and SNORD78 also correlated with altered 2′-O-Me activities within 28S rRNA at corresponding sites (28S-Gm3878, 28S-Gm4593) that contribute to cell fate by influencing downstream target protein synthesis and translation fidelity in proteins such as EIF4A3 and LAMC2. We also observed that these regulation patterns can be regulated by ZFAS1. Based on these observations, our data provide new insights into the involvement of lncRNA in the C/D box snoRNAs-mediated 2′-O-Me modification field, ultimately providing a solid basis for future development and for further understanding of pathological properties of post-transcriptional RNA alterations, particularly in cancers.

In summary, our research provides insights into a novel molecular mechanism of the lncRNA ZFAS1 in the regulation of CRC initiation and pathogenesis. By direct binding to the core RNP NOP58, ZFAS1 promoted SNOR12C/78 snoRNPs complex assembly and 28S rRNA 2′-O-methylation at corresponding sites to substantially influence the RNA stability and translation activity their downstream target genes (e.g., EIF4A3 and LAMC2). This, in turn, mediates CRC cell proliferation promotion and apoptosis inhibition in vitro and in vivo. Our study identified a previously unrecognized signaling axis involving ZFAS1-NOP58-SNORD12C/78-EIF4A3/LAMC2 that functions in CRC development and progression. Therefore, our work sheds new light on the potential applications of lncRNA, snoRNAs, and cellular 2′-O-methylation-dependent translation networks in the prevention and therapy of CRCs.

## Supplementary information

**Additional file 1: Table S1.***Short hairpin RNAs* (*shRNAs*) sequence against ZFAS1. **Table S2.***Short hairpin RNAs* (*shRNAs*) sequence against NOP58. **Table S3.** The reaction assays of qRT-PCR method. **Table S4.** Primers used in qRT-PCR assays. **Table S5.** Probes used in situ hybridization (ISH) assay. **Table S6.** RNA probes used for RNA pull-down assays. **Table S7.** Primers used in site-specific 2′-O-methylation for RTL-P assay. **Table S8.** RTL-P assays for rRNA 2′-O-methylation. **Table S9.** Probes used in site-specific 2′-O-methylation for double-stranded primer based on single-stranded toehold (DPBST) assays. **Table S10.** Double-stranded primer based on single-stranded toehold (DPBST) assays. **Table S11.** Data of LncRNAs cluster in Heat map analysis. **Table S12.** Data of snoRNAs cluster in Heat map analysis. **Table S13.** Data of mRNAs cluster in Heat map analysis. **Table S14.** The enriched target genes intersected by ZFAS1, SNORD12C/78 in GO analysis. **Table S15.** Prognostic information of included colorectal cancer patients (*n* = 157). **Table S16.** Multivariate COX regression analysis of the association of ZFAS1 and NOP58 expression with DFS and OS in CRC patients.

**Additional file 2.** Supplementary materials and methods.

**Additional file 3 Figure S1.** The expression and correlation analysis between ZFAS1 and snoRNP complex based on the TCGA database. **Figure S2.** The (m) RNA expression of ZFAS1, NOP58, SNORD12C, and SNORD78 in CRC tissues and matched tumor-adjacent controls (*n* = 30). **Figure S3.** The correlation analysis of ZFAS1, NOP58, SNORD12C/78 expression in 30 paired CRC and control tissues. **Figure S4.** The effect of ZFAS1 on cell proliferation and apoptosis in CRC cells. **Figure S5.** The correlation of ZFAS1 and NOP58 expression and prognosis evaluation in CRC patient tissues. **Figure S6.** The 2′-O-Me activity levels after interfering ZFAS1 and/or NOP58 expression. **Figure S7.** The 2′-O-Me activities mediated by SNORD12C and SNORD78 after silencing SNORD12C and SNORD78.

## Data Availability

Raw data of RNA deep-sequencing datasets have been deposited in Gene Expression Omnibus under accession number GSE137511 (*https://www.ncbi.nlm.nih.gov/geo/query/acc.cgi?acc=GSE137511*). The authors declare that all the data supporting the findings in this study are available in this study and its Supplementary materials, or are available from the corresponding author through reasonable request.

## Abbreviations

rRNA: Ribosome RNA
2′-O-Me: 2′-O-methylation
CRC: Colorectal cancer
ncRNAs: Non-coding RNAs
snoRNAs: Small nucleolar RNAs
SNORDs: C/D box small nucleolar RNAs
lncRNAs: Long non-coding RNAs
snoRNPs: Small nucleolar ribonucleoprotein complexes
TMA: Tissue microarray
IHC: Immunohistochemistry
ISH: In situ hybridization
ROC curve: Receiver operating characteristic curve
RTL-P: Reverse Transcription at Low deoxy-ribonucleoside triphosphate (dNTP) concentrations followed by polymerase chain reaction (PCR)
DPBST: Double-stranded primer based on single-stranded toehold assay
