# Supplementary material for: Long noncoding RNA ZFAS1 promoting small nucleolar RNA-mediated 2′-O-methylation via NOP58 recruitment in colorectal cancer
Source: Mol Cancer. 2020 May 22;19:95. doi: 10.1186/s12943-020-01201-w (PMC7243338; doi:10.1186/s12943-020-01201-w)
Supplement: Supplementary file 1 — Additional file 1: Table S1.Short hairpin RNAs (shRNAs) sequence against ZFAS1. Table S2.Short hairpin RNAs (shRNAs) sequence against NOP58. Table S3. The reaction assays of qRT-PCR method. Table S4. Primers used in qRT-PCR assays. Table S5. Probes used in situ hybridization (ISH) assay. Table S6. RNA probes used for RNA pull-down assays. Table S7. Primers used in site-specific 2′-O-methylation for RTL-P assay. Table S8. RTL-P assays for rRNA 2′-O-methylation. Table S9. Probes used in site-specific 2′-O-methylation for double-stranded primer based on single-stranded toehold (DPBST) assays. Table S10. Double-stranded primer based on single-stranded toehold (DPBST) assays. Table S11. Data of LncRNAs cluster in Heat map analysis. Table S12. Data of snoRNAs cluster in Heat map analysis. Table S13. Data of mRNAs cluster in Heat map analysis. Table S14. The enriched target genes intersected by ZFAS1, SNORD12C/78 in GO analysis. Table S15. Prognostic information of included colorectal cancer patients (n = 157). Table S16. Multivariate COX regression analysis of the association of ZFAS1 and NOP58 expression with DFS and OS in CRC patients. [file 12943_2020_1201_MOESM1_ESM.pdf]

Additional file 1

Long noncoding RNA ZFAS1 promoting small nucleolar RNA-mediated 2'-O-methylation via NOP58 recruitment in colorectal cancer

This file includes:

- **Table S1**. *Short hairpin RNAs* (*shRNAs*) sequence against ZFAS1
- **Table S2**. *Short hairpin RNAs* (*shRNAs*) sequence against NOP58
- **Table S3.** The reaction assay of qRT-PCR method
- **Table S4.** Primers used in qRT-PCR assay
- **Table S5.** Probes used in situ hybridization (ISH) assay
- **Table S6.** RNA probes used for RNA pull-down assay
- **Table S7.** Primers used in site-specific 2'-O-methylation for RTL-P assay
- **Table S8.** RTL-P assay for rRNA 2'-O -methylation
- **Table S9.** Probes used in site-specific 2'-O-methylation for double-stranded primer based on single-stranded toehold (DPBST) assay
- **Table S10.** Double-stranded primer based on single-stranded toehold (DPBST) assay
- **Table S11.** Data of LncRNAs cluster in the Heat map analysis
- **Table S12.** Data of snoRNAs cluster in the Heat map analysis
- **Table S13.** Data of mRNAs cluster in the Heat map analysis
- **Table S14.** The enriched target genes intersected by ZFAS1, SNORD12C/78 in GO analysis
- **Table S15.** Prognostic information of included colorectal cancer patients (*n*=157)
- **Table S16.** Multivariate COX regression analysis of the association of ZFAS1 and NOP58 expression with DFS and OS in CRC patients

Table S1. ***Short hairpin RNAs (shRNAs)*** **sequence against ZFAS1**

**shRNA**	**Sequence (5'-3')**
ZFAS1-Homo-455	GCCATTCGTTCTTTCGCGTCT
ZFAS1-Homo-525	GCTATTGTCCTGCCCGTTAGA
ZFAS1-Homo-769(shRNA#1)	GATTCAGTCTGCCTTGTAACA
ZFAS1-Homo-884(shRNA#2)	CAAGGTTACTGTATACATAGC

Table S2. ***Short hairpin RNAs (shRNAs)*** **sequence against NOP58**

shRNA	Sequence (5'-3')
NOP58-Homo-386	GCAGAAGCATTAGCAGCATTC
NOP58-Homo-660	GCCTGTCTCGATATAGATTGA
NOP58-Homo-932(shRNA#1)	GCAGCTGCAGAGATATCAATG
NOP58-Homo-1520(shRNA#2)	GGTGACTCCACACTTCCAACC

Table S3. **The reaction assay of qRT-PCR method**

**mRNA and LncRNA**	
**Relative reagents**	**1×system (10μL)**
5×RT Buffer	2 μL
Enzyme Mix	0.5 μL
Primer Mix	0.5 μL
RNA+DEPC H_2O	7 μL (400ng)
**Program: 37 ºC/ 15min→ 98 ºC/ 5min→ Maintain at 4 ºC**	
**snoRNA**	
**Relative reagents**	**1×system(10μL)**
5×Buffer	2 μL
M-MLV	0.3 μL
RII	0.25 μL
dNTP(10mM each)	1 μL
Proprietary RT primer	1 μL
snoRNA+DEPC H_2O	5.45 μL (1000ng)
**Program: 42 ºC/ 60min→ 70 ºC/ 10min→ Maintain at 4 ºC**	
**qPCR**	
**Relative reagents**	**1×system(10μL)**
SYBR	5 μL
ROX	0.2 μL
Forward Primer(10μM)	0.4 μL
Reverse Primer(10μM)	0.4 μL
cDNA	4 μL
**Program: 95 ºC/ 60sec→[95 ºC/15sec→ 60 ºC/15sec→72 ºC/45sec]×40cycles→ Maintain at 4 ºC**	

Table S4. Primers used in qRT-PCR assay

Primer	Sequence (5'-3')
ZFAS1-Forward	GCTATTGTCCTGCCCGTTAG
ZFAS1-Reverse	TCGTCAGGAGATCGAAGGTT
NOP58-Forward	GGCAGCGTGTTCTGATTCTT
NOP58-Reverse	GCCTGCCAGAAGCTGTAGAGT
FBL-Forward	GTGGAGGAGGAGGAAGAGGT
FBL-Reverse	GGCTCCACCATCACATTCTT
NOP56-Forward	CTGGAGGAGCTGACAATGGA
NOP56-Reverse	CACCACACGACTGGAGAAGC
SNU13-Forward	CCTCTGCCATCCTTCATCCT
SNU13-Reverse	TCTCTGTCCTGGCTCCATCA
LAMC2-Forward	CTGGGTTGTGCACATTTCTTT
LAMC2-Reverse	AAATACAGAAGCAAGGCAGCA
EIF4A3-Forward	GCTGCTTGCTCTCGGTGACTAC
EIF4A3-Reverse	GCTTCCTGATGTCCTCGCCAAC
MACC1-Forward	TTCTTTTGATTCCTCCGGTGA
MACC1-Reverse	ACTCTGATGGGCATGTGCTG
CSE1L-Forward	TTAATAGTTCAGTGTATGGCGTTGG
CSE1L-Reverse	AGTCTCCACAGTGTCCGTCAAA
GAPDH-Forward	TTGCCCTCAACGACCCTTT
GAPDH-Reverse	TCCTCTTGTGCTCTTGCTGG

Table S5. Probes used in situ hybridization (ISH) assay

Digoxin-labeled probe	Sequence (5'-3')
ZFAS1-(1)	GGAAC CCGTC GAGCG GTTTG GTGCG TGTGA AGCGC GACAT
ZFAS1-(2)	GGTTA TATAA GGGAG GTTCA GGAAG CCATT CGTTC TTTCG
ZFAS1-(3)	CTACA ACCTT CGATC TCCTG ACGAG TTTAT TGTTG GCCAA

Table S6. RNA probes used for RNA pull-down assay

Probe	Sequence (5'-3')	Modified method
ZFAS1-WT	GATTTTGG**AAGA**GGGAGTCACCACTGGAC	5’ Biotin
ZFAS1-mut	GATTTTGG**TTCT**GGGAGTCACCACTGGAC	5’ Biotin
ZFAS1-antisense	CTAAAACCTTCTCCCTCAGTGGTGACCTG	5’ Biotin

Table S7. Primers used in site-specific 2'-O-methylation for RTL-P assay

Primer	Sequence (5'-3')
SNORD12C-Fu	CCUACUAUCCAGCGAAACCAC
SNORD12C-Fd	AAAGAAGACCCTGTTGAGCTT
SNORD12C-R	TATTCTACACCTCTCATGTCT
SNORD12C-RT	TATTCTACACCTCTCATGTCTCT
SNORD78-Fu	TAATCCTGCTCAGTACGAGA
SNORD78-Fd	CTGTATGTGCTTGGCTGAG
SNORD78-R	ATTCTGACTTAGAGGCGTTCA
SNORD78-RT	CGCTGCCGTATCGTTCGCCTG

Table S8. RTL-P assay for rRNA 2’-O-methylation

Low dNTP reverse transcription reaction	
**Relative reagents**	**1×system (10μL)**
Total RNA	1 μL(100ng)
Specific RT primer(50μM)	1 μL
RNase free H_2O	up to 6 μL
**Program: 70 ºC/10min→ Ice 2min**	
**Add the following reverse transcription reaction solution**	
5×M-MLV Buffer	2 μL
RTase M-MLV(RNase H-)(200U/μL)	0.5 μL
RNase Inhibitor(40U/μL)	0.25 μL
dNTP (low 10μM; high 1mM)	0.5 μL
RNase free H_2O	up to 10 μL
**Program: 42 ºC/60min → 70 ºC/15min → Maintain at 4 ºC**	
**PCR**	
**Relative reagents**	**1×system(10μL)**
10×Ex taq Buffer	2.5μL
Ex taq DNA polymerase(5U/μL)	0.25 μL
dNTP(10mM)	0.5 μL
Forward Primer(50μM)	0.5 μL
Reverse Primer(50μM)	0.5 μL
cDNA	2 μL
RNase free H_2O	up to 25 μL
**Program: 94 ºC/4min→[94 ºC/30sec→ 55 ºC/30sec→72 ºC/1min]×21cycles→ Maintain at 4 ºC**	

Table S9. Probes used in site-specific 2'-O-methylation for Double-stranded primers based on single-stranded toehold (DPBST) assay

BST dsPrimers	Sequence (5'-3')
SNORD12-sense	GCCTCCCACTTATTCTACACCTCTCATGTCTCTTCACCGTGCCAG ACTAGAGTCAAGCTCAACAGGGTCTTCTTTC
SNORD12-antisense	GAAAGAAGACCCTGTTGAGCTTGACTCTAGTCTGGCACGGTGAA GAGACAACTC-C6 spacer
SNORD12C-Forward	CCTACTATCCAGCGAAACCAC
SNORD12C-Reverse	GCCTCCCACTTATTCTACACC
SNORD78-sense	GAGGCGTTCAGTCATAATCCCACAGATGGTAGCTTCGCCCCATT GGCTCCTCAGCCAAGCACATACACCAAA
SNORD78-antisense	ACATTTGGTGTATGTGCTTGGCTGAGGAGCCAATGGGGCGAAGC TACCATGACA-C6 spacer
SNORD78-Forward	GATGTCGGCTCTTCCTATCAT
SNORD78-Reverse	TCTCGTACTGAGCAGGATTAC

Table S10. Double-stranded primer based on single-stranded toehold (DPBST) assay

**Probes pretreatment**	
Probe A and B mix in equal volume	
**Program: 90 ºC/1min → Room temperature 5min**	
**Reverse transcription reaction**	
**Relative reagents**	**1×system (10μL)**
Total RNA	1 μL(100ng)
Specific RT primer(A and B each 10μM)	2 μL
RNase free H_2O	up to 6 μL
**Program: 70 ºC/10min → Ice 2min**	
**Add the following reverse transcription reaction solution**	
5×M-MLV Buffer	2 μL
RTase M-MLV(RNase H-)(200U/μL)	0.5 μL
RNase Inhibitor(40U/μL)	0.25 μL
dNTP(10mM)	0.5 μL
RNase free H_2O	up to 10 μL
**Program: 42 ºC/60min → 70 ºC/15min → Maintain at 4 ºC**	
**qPCR**	
**Relative reagents**	**1×system(10μL)**
TB Green premix Ex TaqII	10μL
Forward Primer(50μM)	0.8 μL
Reverse Primer(50μM)	0.8 μL
ROX(50×)	0.4 μL
cDNA dNTP(10mM)	2 μL
RNase free H_2O	up to 20 μL
**Program: 95 ºC/30sec→[95 ºC/5sec→ 60 ºC/34sec → 95 ºC/ 1min] ×40 cycles → Maintain at 4 ºC**	

Table S11. Data of LncRNAs cluster in the Heat map analysis

Up-regulated		Down-regulated	
**LncRNA**	**Fold Change**	**LncRNA**	**Fold Change**
**ZFAS1**	6.65	RNF138P1	2.21
TERC	3.17	PSMD6-AS2	2.64
TECRP1	2.05	PGM5P2	2.64
SNHG8	3.45	NPY6R	4.92
SNHG17	2.11	NCF1C	2.02
SCARNA9L	3.11	NCF1B	2.27
SCARNA23	3.12	MIR650	4.14
RPS2P7	2.75	MIR628	2.10
RPL13AP20	2.06	MIR490	2.04
RPL12P2	2.05	MIR4521	2.25
RNU5F-1	2.13	MIR4451	2.02
RANP1	2.93	MIR107	2.04
PSAT1P4	2.43	LOC285972	2.37
PROX1-AS1	2.29	LOC285878	4.13
MIR622	2.98	LINC00675	2.64
MIR614	2.42	LINC00641	3.06
MIR3189	4.02	LINC00294	2.24
MIR1206	4.34	IGKV2-29	8.77
MIR1204	3.72	GVINP1	3.54
MGC32805	2.78	FENDRR	4.01
LOC541471	2.56	CMAHP	6.25
LINC00493	2.10	ANKRD36BP2	3.31
LINC00273	2.46	ADAMTS9-AS2	3.18
HSP90AB3P	2.18	ABCC13	2.12
HSD17B7P2	2.14		
FEZF1-AS1	3.69		
FAM83H-AS1	2.16		
CRNDE	3.88		
CCAT1	54.88		
BACE2-IT1	3.05		

Table S12. Data of snoRNAs cluster in the Heat map analysis

**Up-regulated**		**Down-regulated**	
**snoRNA**	**Fold Change**	**snoRNA**	**Fold Change**
SNORD12	9.02	SNORD115-6	3.20
SNORD12B	7.42	SNORD115-5	3.08
**SNORD12C**	5.71	SNORD115-44	2.42
SNORD72	3.99	SNORD115-43	3.38
SNORD70	3.21	SNORD115-42	3.10
SNORD56	2.25	SNORD115-40	2.59
SNORD47	3.74	SNORD115-4	2.31
SNORD27	2.96	SNORD115-39	2.18
SNORD24	2.14	SNORD115-34	5.80
SNORD1C	4.85	SNORD115-24	2.10
SNORD1B	2.65	SNORD115-21	2.96
SNORD19	2.11	SNORD115-16	2.65
SNORD15A	3.10	SNORD115-15	2.50
SNORD14E	2.33	SNORD115-12	3.08
SNORD14D	2.21	SNORD115-11	3.38
SNORD14B	2.89	SNORD115-1	2.47
SNORD87	3.02		
**SNORD78**	6.86		
SNORD75	3.06		
SNORD123	2.39		
SNORD11B	2.15		
SNORA74A	2.30		
SNORA71D	5.83		
SNORA71C	12.02		
SNORA71B	3.27		
SNORA71A	17.16		
SNORA70G	2.03		
SNORA70D	2.02		
SNORA70C	2.22		
SNORA70	2.28		
SNORA68	2.81		
SNORA65	4.82		
SNORA5A	2.70		
SNORA59B	2.68		
SNORA41	2.26		
SNORA32	2.61		
SNORA31	3.90		
SNORA24	2.58		
SNORA23	2.44		

Table S13. Data of mRNAs cluster in the Heat map analysis

Up-regulated		Down-regulated	
**mRNA**	**Fold Change**	**mRNA**	**Fold Change**
**NOP58**	4.22	LARGE	1.54
PAK1IP1	2.47	EIF4E3	2.17
RPL30	2.08	VSIG2	3.19
DDX21	7.19	TMEM63B	2.01
ERP27	3.98	TCF7L2	2.26
MACC1	8.89	NAA60	1.51
ESM1	4.14	ATP5A1	2.04
MYC	6.71	ZYG11B	1.71
CEBPZ	1.70	HSBP1L1	2.50
RPS14	2.13	TLCD2	1.63
ZNF280C	2.44		
MMP10	2.42		
SNRPB2	2.48		
FANCB	2.71		
TTC26	2.59		
RAP2A	2.13		
DIMT1	2.24		
PSMG1	1.85		
RRS1	3.37		
FKBP10	2.13		
TUBG1	1.87		
LAMC2	6.15		
WDR12	2.28		
TRIM27	1.65		
TOMM22	1.70		
MTDH	1.60		
GRIN2B	10.82		
NUP155	3.42		
RPS2	2.39		
GINS1	2.84		
AGO2	2.06		
XPO7	1.65		
LRP8	2.60		
RNF43	12.08		
WDYHV1	1.56		
GRPEL1	1.91		
KCNH8	3.96		
SNRPF	1.61		
WDR74	1.92		
PLEK2	3.03		

ERO1L	4.91
RPL39	3.22
MAPRE1	2.00
ADNP	1.75
IER3	10.30
SKP2	2.75
NAT10	2.48
ACOT9	1.80
RPS27A	1.69
NHP2L1	1.47

Table S14. The enriched target genes intersected by ZFAS1, SNORD12C/78 in GO analysis

Intersected target genes			
PRPF40A	LRP8	CIRH1A	RPS25
NUP35	CAD	PPAT	C10orf113
POLR2G	SKP2	POLA1	CEBPZ
GNL3	PSMG1	ENO1	GRHL3
SLCO4A1	WDR43	RAP2A	ANAPC1
MYO1B	ASUN	C11orf1	RPL39
G3BP1	FOPNL	RPS2	RBM3
NUP155	RPL28	SNAI1	ACOT9
LMO7	QTRT1	BRWD3	IL1RAP
TOP1MT	MPP6	SOX9	CSE1L
POLR1C	EIF2S3L	SHROOM4	RNF43
TRIO	PPP2R2A	EIF2S3	WDYHV1
CSTF1	EIF3M	RPL35A	E2F5
RBM12	GNB2L1	MORF4L2	LARS2
PNPT1	RPS27A	MOCS3	CBWD5
IFITM3	RPS3	RBMX	HKDC1
RPL36A	RAD18	PRPF6	PSME3
CMSS1	WDR75	ANO9	XPOT
PTCD3	EIF2A	PAICS	UTP14A
NAT10	TTI1	FKBP10	DKC1
DARS	MAPRE1	NIT2	PAK1IP1
PLEK2	TUBG1	RPS7	NMD3
URB1	CBWD3	LAMC2	GART
PSMA3	ERO1L	NONO	RSL1D1
GINS1	XPNPEP3	RPL37A	PPA1
TMEM182	MTDH	SIM2	MYC
RPL29	HSP90AB1	IGBP1	SNX20
GRPEL1	ABHD12	GRIN2B	TOMM22
RPS20	SNRPF	ZCCHC7	SULT2B1
KCNH8	RPL18	PABPC1	RPS14
IMPDH2	TRBV6-8	WDR36	RPL30
RPS10	HPS4	POFUT1	TTC27
DDX31	NEBL	XPO4	DIMT1
RPP40	MACC1	RUVBL1	NUDCD1
POLR1B	BHLHE40	RPL11	RCC1
PHKA2	MAK16	INTS10	G6PC3
KIAA0020	PUS1	RPL8	GEMIN5
GTPBP4	RPS13	LRP4	CCT6A
GTF3C3	WDR12	TRIM27	SFXN3
AP3M2	MRE11A	GABRE	XPO7
ZNF280C	GSTP1	GRPEL2	IKBKAP

CBWD6	DDX21	C11orf49	FANCB
WDR74	NOP58	MKI67IP	TARBP1
SLC25A32	WRN	EPHX4	RBL1
ARMC1	SF3A3	QRSL1	AMER1
ADAT2	RIPK2	RRS1	RPF2
SLC15A4	DDX10	ADNP	MTERFD1
YEATS2	ALYREF	STC2	TTC26
ERP27	MFSD12	SNRPB2	HSP90AA1
PUS7	PPRC1	RPL31	CCT7
SH2D4A	MTFR1		

Table S15. Prognostic information of included colorectal cancer patients (*n*=157)

patient	ZFAS1 (high or low)	NOP58 (high or low)	EIF4A3 (high or low)	LAMC2 (high or low)	DFS state	DFS (months)	OS state	OS (months)
1	0	0	0	1	dead	25	dead	25
2	1	0	1	0	dead	20	dead	20
3	0	1	0	1	alive	55	alive	55
4	1	0	1	0	alive	98	alive	98
5	1	1	1	1	alive	55	alive	55
6	1	0	1	1	dead	11	dead	11
7	1	1	0	0	alive	98	alive	98
8	1	1	0	1	dead	25	dead	25
9	0	0	1	1	alive	110	alive	110
10	1	0	1	1	alive	53	alive	53
11	1	1	1	1	dead	36	dead	36
12	0	0	1	0	alive	72	alive	72
13	0	1	0	0	dead	30	dead	30
14	1	1	1	0	alive	74	alive	74
15	0	0	0	0	alive	92	alive	92
16	0	1	0	1	dead	75	dead	75
17	0	0	0	0	dead	63	dead	63
18	0	0	0	0	dead	24	dead	24
19	0	1	1	0	dead	90	alive	97
20	0	1	1	0	alive	89	alive	89
21	1	1	1	0	dead	24	dead	24
22	1	1	1	1	alive	66	alive	66
23	0	0	1	1	alive	104	alive	104
24	0	1	1	1	alive	78	alive	78
25	0	0	1	0	alive	55	alive	55
26	0	1	0	0	dead	39	dead	39
27	0	0	0	0	dead	26	dead	26

28	0	1	0	1	dead	28	dead	24
29	1	0	1	1	dead	75	dead	82
30	0	0	1	1	dead	50	dead	50
31	0	0	0	1	dead	75	dead	75
32	0	1	0	1	alive	102	alive	102
33	0	0	0	0	dead	2	dead	2
34	0	0	0	0	alive	87	alive	87
35	0	1	1	1	dead	6	dead	15
36	1	0	0	1	alive	105	alive	105
37	1	1	1	0	dead	24	dead	24
38	0	0	0	0	dead	24	dead	30
39	1	1	1	0	dead	10	dead	10
40	1	0	0	1	dead	8	dead	8
41	1	1	0	1	dead	26	dead	26
42	0	1	0	0	dead	11	dead	11
43	0	0	1	0	dead	42	dead	42
44	1	0	1	0	dead	38	dead	38
45	0	0	0	1	dead	20	alive	28
46	1	1	0	1	dead	87	alive	93
47	1	0	0	1	dead	27	dead	27
48	1	0	1	1	alive	90	alive	90
49	0	0	0	0	alive	105	alive	105
50	1	0	1	1	alive	101	alive	101
51	0	0	1	1	dead	30	dead	37
52	1	1	0	1	alive	95	alive	95
53	1	0	1	0	alive	103	alive	103
54	1	0	0	0	dead	24	dead	24
55	1	0	1	0	alive	88	alive	88
56	1	0	0	1	dead	12	dead	12
57	0	0	1	0	dead	25	dead	25

58	0	0	0	0	dead	16	dead	16
59	0	1	0	1	alive	88	alive	88
60	1	0	1	1	alive	82	alive	82
61	0	1	1	1	dead	24	dead	24
62	0	0	1	1	alive	103	alive	103
63	0	1	0	1	alive	51	alive	51
64	0	0	0	1	dead	24	dead	24
65	1	0	0	0	dead	28	dead	28
66	0	1	1	1	dead	52	dead	52
67	0	0	0	0	dead	50	dead	50
68	0	1	1	0	alive	0	alive	0
69	0	0	0	0	dead	24	dead	24
70	1	0	1	1	alive	94	alive	94
71	0	0	0	0	alive	66	alive	66
72	1	1	1	0	dead	13	dead	13
73	1	0	1	1	dead	24	dead	24
74	0	1	0	1	dead	68	alive	73
75	0	0	1	1	dead	76	dead	76
76	0	1	0	0	dead	15	dead	15
77	0	1	0	0	dead	14	dead	14
78	0	0	1	1	dead	40	dead	40
79	0	1	0	0	alive	102	alive	102
80	0	0	0	0	dead	60	alive	64
81	0	0	0	0	alive	106	alive	106
82	1	1	1	1	dead	13	dead	13
83	0	0	0	0	alive	36	alive	36
84	0	1	0	1	dead	50	dead	50
85	0	0	0	1	dead	24	dead	24
86	0	1	0	1	alive	80	alive	80
87	0	0	0	0	dead	30	dead	30

88	0	1	0	0	alive	102	alive	102
89	0	0	1	0	dead	16	dead	24
90	1	1	1	0	dead	1	dead	1
91	1	1	0	0	dead	40	dead	40
92	0	0	0	0	dead	19	dead	25
93	1	1	1	0	dead	25	dead	25
94	0	0	0	1	dead	15	dead	20
95	0	0	0	1	dead	12	dead	18
96	0	1	1	1	dead	25	dead	25
97	0	1	1	0	alive	105	alive	105
98	0	0	0	0	dead	36	dead	36
99	0	0	0	0	dead	92	alive	100
100	0	0	0	0	alive	67	alive	67
101	0	1	1	1	dead	20	dead	20
102	0	1	0	0	dead	3	dead	15
103	0	0	0	0	dead	25	dead	25
104	0	0	0	1	dead	52	dead	52
105	1	1	1	0	alive	36	alive	36
106	0	1	0	0	dead	44	dead	50
107	0	0	0	1	alive	63	alive	63
108	0	1	1	1	dead	55	alive	60
109	1	1	1	1	alive	89	alive	89
110	0	0	0	1	dead	20	dead	20
111	0	1	0	1	dead	76	dead	76
112	1	0	1	0	dead	58	alive	58
113	0	1	1	0	dead	18	dead	24
114	0	0	0	1	dead	16	dead	16
115	1	1	1	1	dead	23	dead	23
116	0	1	1	1	alive	105	alive	105
117	1	0	1	0	dead	30	dead	30

118	0	1	0	0	dead	16	dead	16
119	0	0	0	0	dead	15	dead	15
120	0	0	1	0	dead	26	dead	26
121	0	0	1	0	dead	48	alive	54
122	1	0	1	1	dead	30	dead	30
123	0	0	0	0	dead	6	dead	6
124	0	1	1	0	dead	80	dead	80
125	0	0	0	0	dead	20	dead	20
126	1	0	0	0	dead	36	dead	36
127	0	0	0	1	alive	98	alive	98
128	1	1	1	1	alive	76	alive	76
129	0	0	0	0	dead	36	dead	36
130	1	1	0	1	dead	24	dead	30
131	1	1	0	1	dead	88	alive	96
132	0	1	0	0	dead	30	dead	30
133	0	0	0	0	dead	62	dead	62
134	1	0	0	1	dead	98	dead	98
135	0	1	1	1	dead	55	dead	61
136	1	0	1	0	dead	24	dead	24
137	0	1	1	0	dead	36	dead	36
138	1	0	0	0	dead	13	dead	13
139	1	0	0	0	dead	37	dead	37
140	0	0	1	0	alive	13	alive	13
141	0	0	0	0	dead	37	dead	37
142	1	1	1	0	dead	14	dead	14
143	0	0	1	0	dead	75	dead	75
144	1	1	1	1	dead	20	dead	26
145	0	0	0	0	dead	30	dead	30
146	0	0	1	1	alive	89	alive	89
147	1	0	0	0	dead	26	dead	26

148	1	1	0	1	alive	63	alive	63
149	0	0	0	1	dead	24	dead	24
150	1	0	0	0	dead	24	dead	24
151	0	1	0	0	dead	20	dead	20
152	1	1	1	0	dead	30	dead	36
153	0	0	0	0	alive	55	alive	55
154	1	1	1	1	dead	24	dead	24
155	0	1	1	1	alive	55	alive	55
156	0	0	1	0	dead	40	dead	40
157	0	0	0	0	dead	19	dead	19

Table S16. Multivariate COX regression analysis of the association of *ZFAS1* and *NOP58* expression with DFS and OS in CRC patients

**Variables**	**DFS**				**OS**			
	**Total *n***	**Events n (%)**	**Adjusted HR(95%CI)**	***P***	**Total *n***	**Events n (%)**	**Adjusted HR(95%CI)**	***P***
**All patients**								
ZFAS1 expression								
Low	100	63(63.0)	1(reference)	-	100	56(56.0)	1(reference)	-
High	57	46(80.7)	1.762(1.196-2.596)	**0.004**	57	43(75.4)	1.815(1.212-2.718)	**0.004**
NOP58 expression								
Low	90	55(61.1)	1(reference)	-	90	48(53.3)	1(reference)	-
High	67	54(80.6)	1.691(1.156-2.473)	**0.007**	67	51(76.1)	1.727(1.160-2.573)	**0.007**

Abbreviations: 95%CI, 95% confidence interval; DFS, Disease-free survival; OS, Overall survival.
P values, Adjusted HR (95%CI) were assessed using multivariate Cox regression analysis adjusted for age, differentiation.
