# Supplementary material for: Long noncoding RNA ZFAS1 promoting small nucleolar RNA-mediated 2′-O-methylation via NOP58 recruitment in colorectal cancer
Source: Mol Cancer. 2020 May 22;19:95. doi: 10.1186/s12943-020-01201-w (PMC7243338; doi:10.1186/s12943-020-01201-w)
Supplement: Supplementary file 2 — Additional file 2. Supplementary materials and methods. [file 12943_2020_1201_MOESM2_ESM.pdf]

Additional file 2

Materials and Methods

Collection of tissue specimen

In this study, human tissue samples were obtained from 157 patients with colorectal cancer, who underwent surgical treatment at the Department of General Surgery of the First Hospital of China Medical University, Department of Medical Oncology of Cancer Hospital of China Medical University between September 2014 and September 2015. This study was approved by the Medical Ethics Committee of China Medical University. All enrolled patients signed the written informed consent form according to the relevant regulations. The tissues of CRC and matched adjacent-tumor controls were snap frozen immediately in liquid nitrogen after separated and stored at -80 ℃ before using.

The inclusion criteria for CRC patients were: 1) availability of complete clinical data and follow-up status; 2) patients with clinical stage II, III and IV; 3) patients who underwent FOLFOX, XELOX chemotherapy. The exclusion criteria include: 1) incomplete clinical data; 2) tissue samples were not available; 3) patients who only underwent radiation therapy; 4) other types of cancer, and cancers with unknown primary sites.

The clinical parameters were collected including age, gender, first-degree family history of CRC, smoking status, tumor size, tumor differentiation, pathological grade, lymph-node metastases from the interviewer administered health risk questionnaires or medical records. The patients were administered chemotherapy treatment for at least 2~3 cycles, and followed up for each month until an occurrence of recurrence and/or death. BMI was calculated from self-reported height and body weight, equal to the body weight (kg)/height^2 (m^2). Tumor differentiation and pathological grade for CRC were performed according to the World Health Organization (WHO) criteria.

LncRNAs and snoRNAs microarray assay

In this study, GeneChip® Human Transcriptome Array 2.0 (HTA2.0, Affymetrix, USA) was selected, the microarray hybridization, and data acquisition were explored by Shanghai OE Biotech Technology Co, Ltd (Shanghai, China) according to the manufacturer's instructions. Total RNA was isolated with Trizol from included four pairs of CRC tissue samples and matched adjacent-tumor normal controls by the NanoDrop ND-2000 (Thermo Scientific) and the RNAs integrity was assessed using Agilent Bioanalyzer 2100 (Agilent Technologies).

The sample labeling, microarray hybridization and washing were performed based on the manufacturer's standard protocols. Briefly, total RNAs were transcribed to double strand cDNAs and then synthesized cRNAs. Next, 2nd cycle cDNAs were synthesized from cRNAs. Followed fragmentation and biotin labeling, the 2nd cycle cDNAs were hybridized onto the microarray. After washing and staining, the arrays were scanned by the Affymetrix Scanner 3000.The standardization of RNA quality control (QC) was assessed based on RNA integrity number (RIN) by using electrophoretic separation on microfabricated chips, then separated and subsequently detected via laser induced fluorescence detection.

This HAT 2.0 designed array contains more than 6.0 million distinct probes covering coding and non-coding transcripts.70% of the probes on this array cover exons for coding transcripts, and the remaining 30% of probes on the array cover exon-exon splice junctions and non-coding transcripts. The array covered more than 285,000 full-length transcripts, more than 245,000 coding transcripts, 40,000 non-coding transcripts and 339,000 probes covering exon-exon junctions of the human genomes. Most of the databases such as *Ensembl*, *UCSC*, *NONCODE*, *RefSeq*, *lncRNAdb*, *Vertebrate Genome Annotation* (Vega), *Mammalian Gene Collection* (MGC), *Human Body Map lincRNAs*, as well as related literatures were used to annotate the determined transcripts.

Affymetrix GeneChip Command Console (version 4.0, Affymetrix) software was used to extract raw data. Next, Expression Console (version1.3.1, Affymetrix) software offered RMA normalization for both gene and exon level analysis. Then the gene expression analysis and alternative splice analysis proceeded separately. The data were analyzed with Robust Multichip Analysis (RMA) algorithm using Affymetrix default analysis settings and global scaling as normalization method. Values presented are log 2 RMA signal intensity. Heat maps representing differentially regulated genes were generated using Cluster 3.0. These microarray data had been deposited on GEO public database under the accession number GSE 137511.

Gene expression analysis

Genesrping software (version 13.1; Agilent Technologies) was employed to perform the raw data analysis. Deferentially expressed genes were then identified through fold change as well as *P* value calculated with *t*-test. The threshold of up- and down-regulated genes was set at fold change ≥ 2.0 and *P* value ≤ 0.05. Afterwards, gene ontology (GO) enrichment analysis and Kyoto Encyclopedia of Genes and Genomes (KEGG) analysis were applied to determine the roles of these deferentially expressed mRNAs played in these GO terms or pathways. Finally, Hierarchical Clustering was performed to display the distinguishable genes' expression pattern among the included 8 samples.

Cell transfection

Plasmid extraction kit was purchased from Sangon Biotech (Shanghai, China).The short hairpin RNA (*shRNA*) for knockdown ZFAS1 (*shZFAS1#1*, *shZFAS1#2*), the negative control (*shNC*) were obtained from GenePharma (Shanghai, China). The *pcDH-ZFAS1-WT*, *pcDH-ZFAS1-Mut*, *pcDH- NOP58-WT*, and *pcDH*-NOP58-Mut were purchased from Genewiz (Suzhou, China). Both of SNORD12C and SNORD78 inhibitors and the negative controls were provided by Ribobio (Guangzhou, China). All of

the *shRNA* nucleotide sequences and the plasmid nucleotide sequences for overexpressing ZFAS1, NOP58 plasmids sequence were listed in Table S1 and Table S2. Cells were plated on 6-well plates to 60%-70% confluence and transfected with 1μg/mL Lipofectamine 3000 (Invitrogen, Carlsbad, CA, USA) according to the manufacturer's instructions.

Reverse-transcription PCR (RT-PCR) assay

Total RNA were isolated from paired CRC tissues or CRC cells using TRIzol reagent according to the manufacturer's instructions. The synthesis of cDNA is conducted from the total RNA using PrimeScript™ RT-PCR Kit (Takara, Japan). The condition of RNA reverse transcription was performed at 65 ℃ for 5 min, and 4 ℃ for 5 min for denaturation, and then PrimeScript RTase and RNase Inhibitor were added for 30 ℃ for 10 min, 42 ℃ for 15 min, 95 ℃ for 5 min, and 4 ℃ for 5 min for reverse transcription. Thereafter, the RT-PCR reaction was performed at (94 ℃ for 30 sec, 60 ℃ for 30sec, 72 ℃ for 1 min) for 30 cycles and 4 ℃ for 5 min. PCR products were separated on a 1.5% agarose gel. All primers were obtained from Sangon Biotech (Shanghai, China).

Quantitative Real-tmie PCR (qPCR) assay

Quantitative real-time PCR (qPCR) were determined using SYBR Green I mix (Toyobo, Japan) in triplicate. The mRNA expression was normalized to reference genes GAPDH and/or U6. The primers used for qPCR were listed in Table S3-Table S4. qPCR was conducted on an Applied Biosystems 7500HT Real-Time PCR System. All oligonucleotide primers were obtained from Sangon Biotech (Shanghai, China). The housekeeping genes, *GAPDH*, and *U6* were used as loading controls. Fold changes were calculated relative to housekeeping genes and normalized to the median value of the benign samples.

Tissue Microarray (TMA) construction

TMA was performed as previously described with brief modification [1, 2]. TMAs comprised of surgical pathology samples from 157 patients with clinically localized colorectal cancer were constructed using tumor tissues and matched adjacent-tumor normal specimens. Paraffin-embedded blocks were prepared by reviewing of the hematoxylin and eosin-stained slides. Tissue cores (0.6 mm in diameter) were removed using a hollow needle from the regions of representative paraffin-embedded tissues. Three tissue cores were selected from FFPE tissue blocks for each included patient sample. Fifty-five of tissues cores are then arrayed into a new recipient paraffin block in a precisely spaced and array pattern by using the Organization Microarrayer (Pathology Devices, USA). Detailed clinical data for this cohort are updated and maintained on a secure relational database.

Evaluation of IHC assay and ISH assay

The results of IHC and ISH staining was evaluated by two pathologists blinded to the experimental conditions. The intensity of immunoreactivity of IHC and specific RNA ISH signal was identified as brown, punctate dots, and expression level was scored as follows: 0 for no staining, 1 for weak staining, 2 for moderate staining, and 3 for strong staining. The proportion of tumor cells was calculated as the percentage of the staining positive cells over the total tumor cells. Five sections were selected from each sample. For each tissue core, a cumulative ISH product score was calculated as the sum of the individual products of the expression level (0 to 3) and percentage of cells (0 to 100), and the total range is from 0 to 300. The scores was assigned by using 10% increments (0%, 10%, 20%, … 300%). The above score for each sample was used for assessing cutoff value for evaluating the expression level (Low or High expression), using receiver operating characteristic curve (ROC), and the optimal cutoff value was obtained based on the maximum of *AUC* (area under the curve), the minimum of sum of sensitivity and 1-specificity for each clinical variable under this included cohort.

Flow cytometry assays

Cells treated with different conditions as indicated were harvested and washed twice with cold 1×PBS. For cell cycle arrest analysis, cells were fixed with 70% ethanol and stored at 4 ℃ overnight. After rehydration with PBS, cells were treated with 20 μl of RNase A (2μg/ml), and incubated at 37°C for 30 min. Cells were then stained with propidium iodide (PI, 50 μg/ml) for 1 h at 4°C. For cell apoptosis analysis, cells were resuspended with 100μL of 1× Annexin V binding buffer, and incubated with 5μl of Annexin V-PE for 15 min and 5μl of 7-AAD for 5 min in a darkroom at room temperature. Finally, cells were analyzed by FACScalibur flow cytometer (BD, USA).

RNA pull-down assay

HEK293T cells with or without ZFAS1 knockdown seeded in 10 cm dish at 70-80% confluency were harvested by trypsinization. Nuclear extraction was isolated by 500 μl 1× hypotonic buffer and 10% NP-40. 40μM of ZFAS1-wild biotin-labeled probe, ZFAS1-mutant biotin-labeled probe, ZFAS1-antisense probe (negative control) were conjugated to Streptavidin agarose resin beads (Thermo Fisher Scientific) by incubation for 4 hours at 4 ℃, respectively, followed by 3wash and incubation with pre-cleared nuclear extraction in 1× binding buffer (pH 7.5) [20 mM Tris, 200 mM NaCl, 6 mM EDTA, 5 mM potassium fluoride, 5 mM glycerophosphate, 2ug/ml apvotinin] at 4 ℃ overnight. After washing with 1× binding buffer for three times, followed by protein isolation with 40μl 1×SDS protein lysis at 95 ℃ for 10 min and 13000g centrifuged for 10 min. Input and co-immunoprecipitated proteins were analyzed by SDS-PAGE separation, and the expression level of NOP58 were measured with GAPDH as internal control.

Statistical analysis

All of the statistical analysis was employed using SPSS 19.0 software package (SPSS Inc. Chicago, USA), and GraphPad Prism 7.0 software (GraphPad, USA). The co-expressed genes were compared and classified by cluster analysis. *Wilcoxon or Welch's T*-test used to compare the significance of the

differences in the expression of indicators, presented as mean ± standard deviation (*s.d.*) or median (quartile). The correlation between the lncRNA, snoRNAs and proteins were analyzed by linear regression analysis. Associations between indicators expression and clinicopathological parameters in CRC patients were analyzed using Pearson χ^2 or Fisher's exact test. Data from RT-PCR, qPCR, cell proliferation, colony assay, Flow cytometry assay, RNA pull-down assay and xenograft mice *in vivo* were analyzed using Student's *t*-test. The survival curves were generated by using the Kaplan-Meier method, Log-rank test, and univariate Cox proportional hazard regression analysis were used to estimate the associations of the Disease-free survival (DFS) or Overall survival (OS) with ncRNA or protein expression. $P<0.05$ was considered statistically significant.

Reference

1. Lu T, Wu B, Yu Y, Zhu W, Zhang S, Zhang Y, Guo J, Deng N: **Blockade of ONECUT2 expression in ovarian cancer inhibited tumor cell proliferation, migration, invasion and angiogenesis**. *Cancer Sci* 2018, **109**(7):2221-2234.
2. Werner TA, Forster CM, Dizdar L, Verde PE, Raba K, Schott M, Knoefel WT, Krieg A: **CXCR4/CXCR7/CXCL12 axis promotes an invasive phenotype in medullary thyroid carcinoma**. *Br J Cancer* 2017, **117**(12):1837-1845.
