# Supplementary material for: Long noncoding RNA ZFAS1 promoting small nucleolar RNA-mediated 2′-O-methylation via NOP58 recruitment in colorectal cancer
Source: Mol Cancer. 2020 May 22;19:95. doi: 10.1186/s12943-020-01201-w (PMC7243338; doi:10.1186/s12943-020-01201-w)
Supplement: Supplementary file 3 — Additional file 3 Figure S1. The expression and correlation analysis between ZFAS1 and snoRNP complex based on the TCGA database. Figure S2. The (m) RNA expression of ZFAS1, NOP58, SNORD12C, and SNORD78 in CRC tissues and matched tumor-adjacent controls (n = 30). Figure S3. The correlation analysis of ZFAS1, NOP58, SNORD12C/78 expression in 30 paired CRC and control tissues. Figure S4. The effect of ZFAS1 on cell proliferation and apoptosis in CRC cells. Figure S5. The correlation of ZFAS1 and NOP58 expression and prognosis evaluation in CRC patient tissues. Figure S6. The 2′-O-Me activity levels after interfering ZFAS1 and/or NOP58 expression. Figure S7. The 2′-O-Me activities mediated by SNORD12C and SNORD78 after silencing SNORD12C and SNORD78. [file 12943_2020_1201_MOESM3_ESM.pdf]

Additional file 3

Long noncoding RNA ZFAS1 promoting small nucleolar RNA-mediated 2'-O-methylation via NOP58 recruitment in colorectal cancer

This file includes:

- **Fig. S1** The expression and correlation analysis between ZFAS1 and snoRNP complex based on the TCGA database
- **Fig. S2** The (m)RNA expression of ZFAS1, NOP58, SNORD12C, and SNORD78 in CRC tissues and matched tumor-adjacent controls (n = 30)
- **Fig. S3** The correlation analysis of ZFAS1, NOP58, SNORD12C/78 expression in 30 paired CRC and control tissues
- **Fig. S4** The effect of ZFAS1 on cell proliferation and apoptosis in CRC cells
- **Fig. S5** The correlation of ZFAS1 and NOP58 expression and prognosis evaluation in CRC patient tissues
- **Fig. S6** The 2'-O-Me activity levels after interfering ZFAS1 and/or NOP58 expression
- **Fig. S7** The 2'-O-Me activities mediated by SNORD12C and SNORD78 after silencing SNORD12C and SNORD78

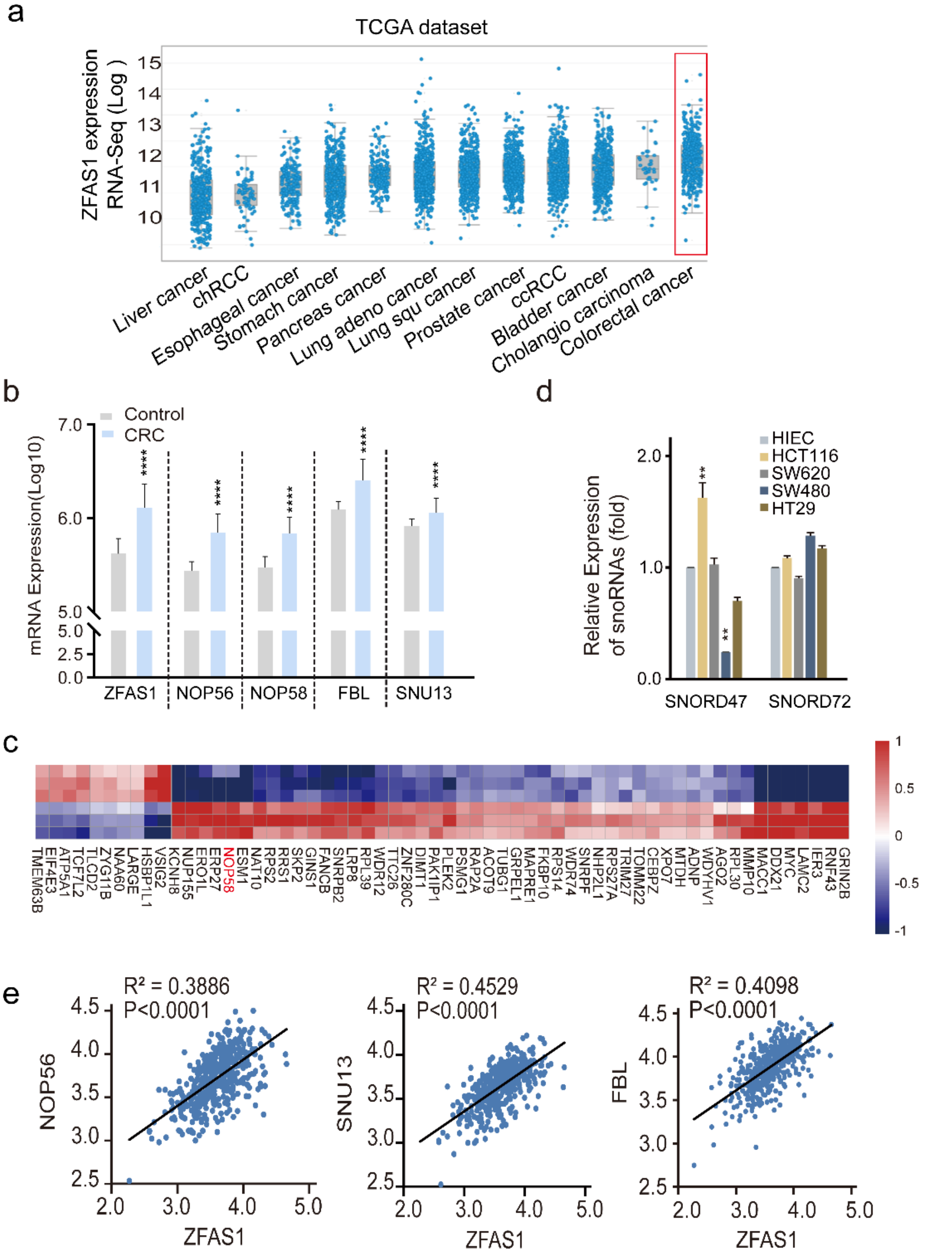

Fig. S1 The expression and correlation analysis between ZFAS1 and snoRNP complex based on the TCGA database

a, RNA-seq analysis illustrating the log 2 gene expression of ZFAS1 in a variety of tumor tissues based on the TCGA data (http://www.cbioportal.org/). **b**, The relative expression of ZFAS1 and its correlated snoRNPs including NOP56, NOP58, FBL, SNU13 in CRC patients tissues (*n* = 638) and the normal controls (*n* = 51) based on the TCGA data. **c**, The hierarchical cluster heat map of log 2 gene expression illustrating the most differential expressed mRNAs in CRC patient tissues vs. paired adjacent tumor control tissues (*n*=3), selected up-regulated or down-regulated genes ($P<0.05$). Red in heat map denotes up-regulation. Blue denotes down-regulation. **d**, The expression levels of SNORD47 and SNORD72 in normal intestinal epithelial HIEC cell and CRC cells including HCT116, SW620, SW480, and HT29 detected by qRT-PCR assays. **e**, The linear correlation analysis representing the relationship of the snoRNP proteins such as SNU13, FBL and NOP56 expression levels with ZFAS1 upon TCGA CRC dataset.

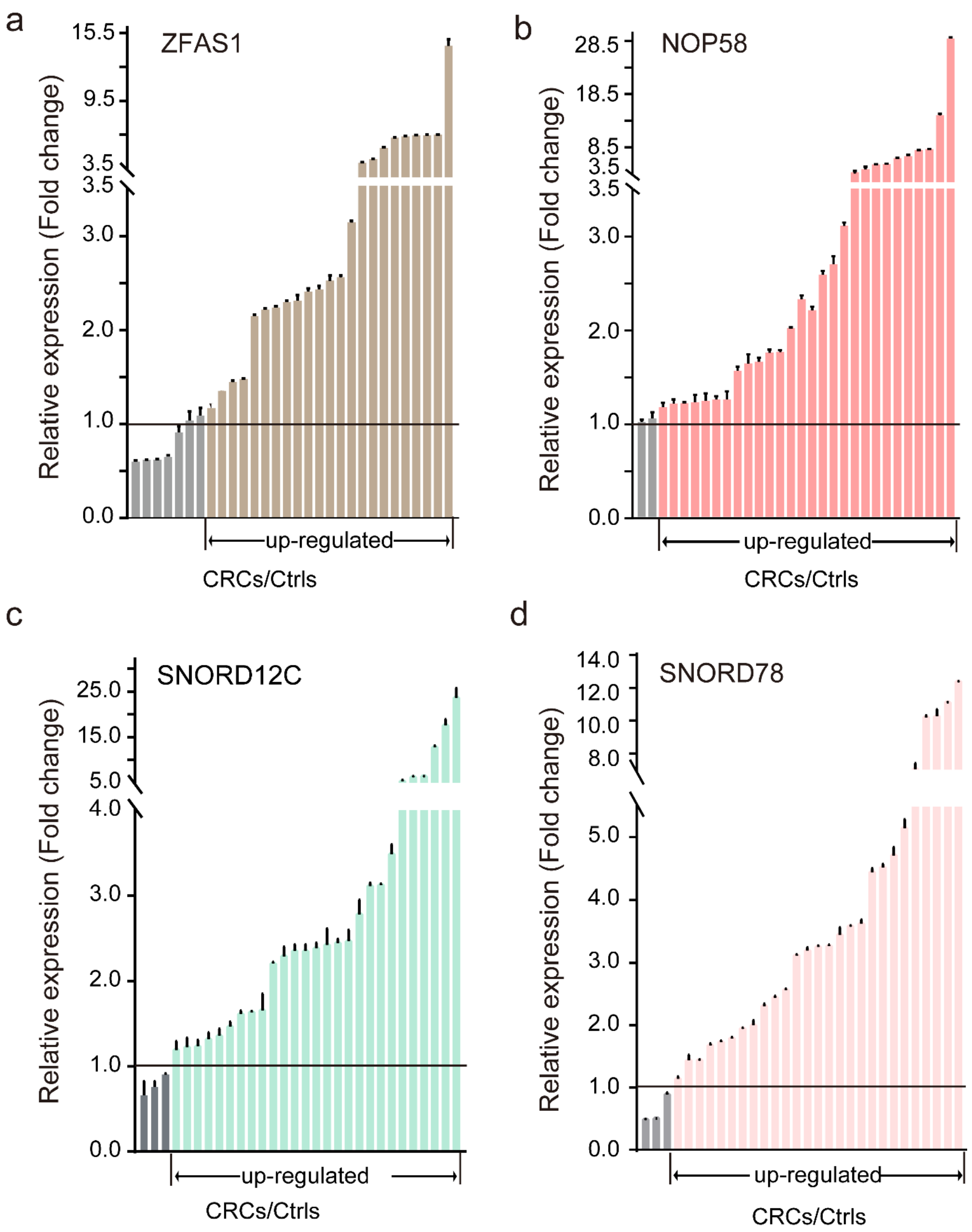

Fig. S2 The (m)RNA expression of ZFAS1, NOP58, SNORD12C, and SNORD78 in CRC tissues and matched tumor-adjacent controls (*n* = 30)

a, b, c, and d, The expression levels of screened ZFAS1(a), NOP58(b), SNORD12C(c), and SNORD78 (d) in 30 pairs of CRC tissues and adjacent-tumor control tissues detected by qRT-PCR assays.

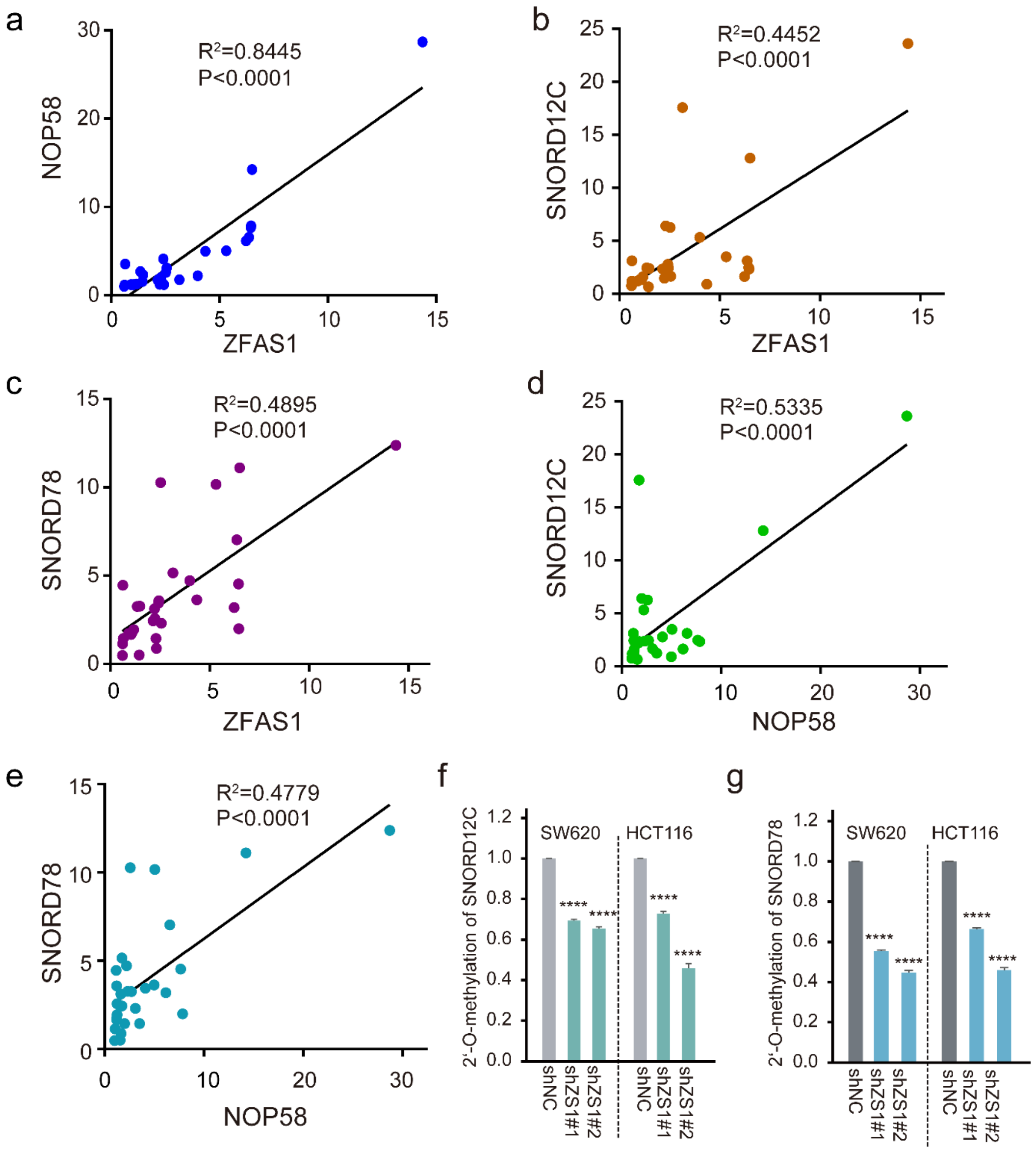

Fig. S3 The correlation analysis of ZFAS1, NOP58, SNORD12C/78 expression in 30 paired CRC and control tissues

The correlation analysis of ZFAS1 and NOP58(**a**), ZFAS1 and SNORD12C(**b**), ZFAS1 and SNORD78(**c**), NOP58 and SNORD12C(**d**), NOP58 and SNORD78(**e**) in 30 pairs of CRC tissues and adjacent-tumor control tissues. **f, g,** The histogram of 2'-O-methylation activities mediated by SNORD12C (f) and SNORD78 (g) after knockdown ZFAS1 in SW620 and HCT116 cells.

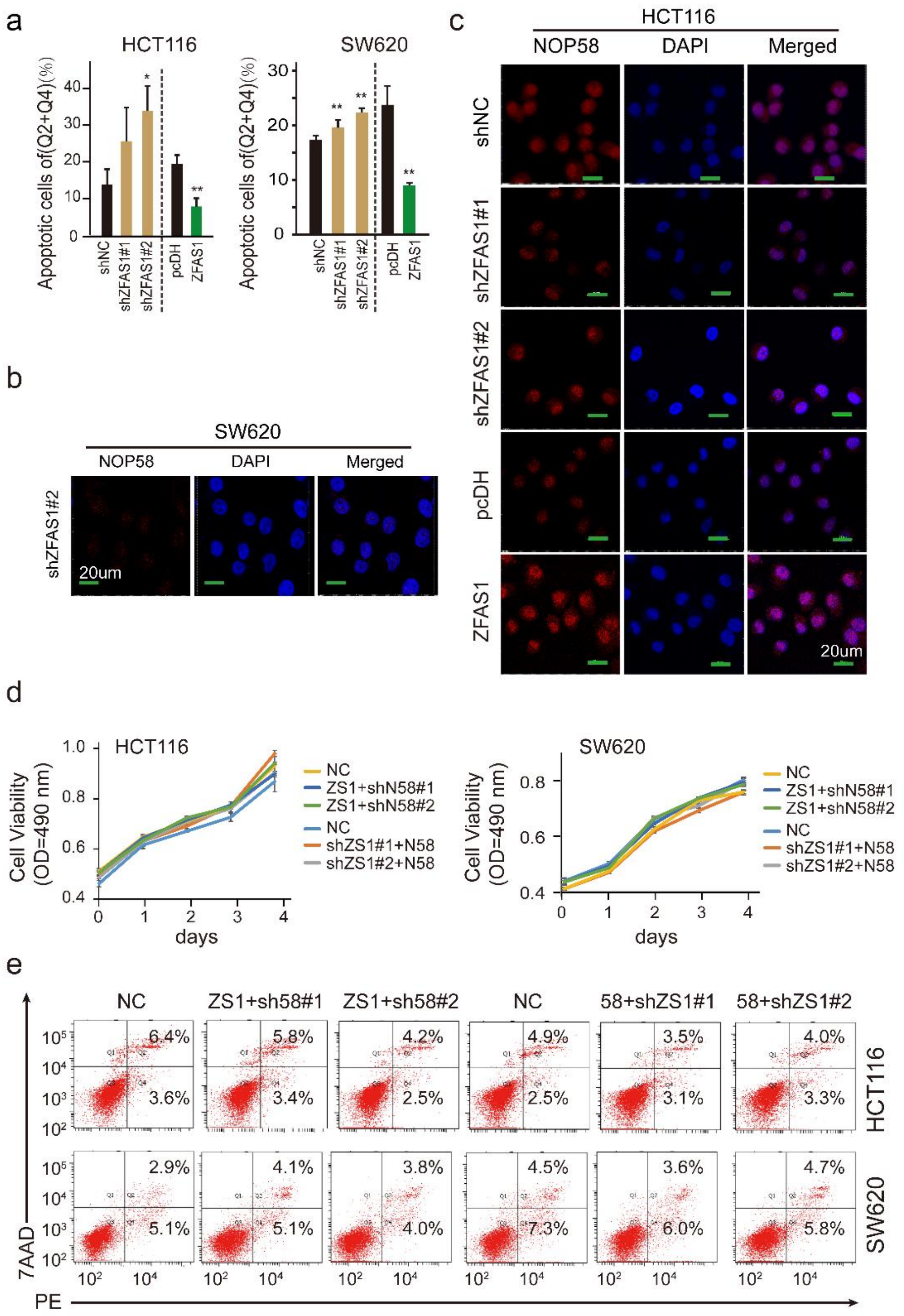

Fig. S4 The effect of ZFAS1 on cell proliferation and apoptosis in CRC cells

a, The histogram of the cell apoptosis percentage upon ZFAS1 overexpressing or silencing in HCT116 and SW620 cells detected by Flow cytometry. **b**, The NOP58 protein expression level was determined after ZFAS1 knockdown by IF assays in SW620 cells. **c**, The NOP58 protein expression level was determined after overexpressing or knockdown ZFAS1 by IF in HCT116 cells. Scale bar = 5μm. **d**, The cell proliferation abilities upon co-transfected with ZFAS1 and NOP58 in HCT116 and SW620 cells. n=3 independent experiments. **e**, The percentage (%) of cell apoptosis was detected based on co-transfected with ZFAS1 and NOP58 in HCT116 and SW620 cells by Flow cytometry.*, P <0.05; **, P <0.01; ***, P <0.001; **** P <0.0001

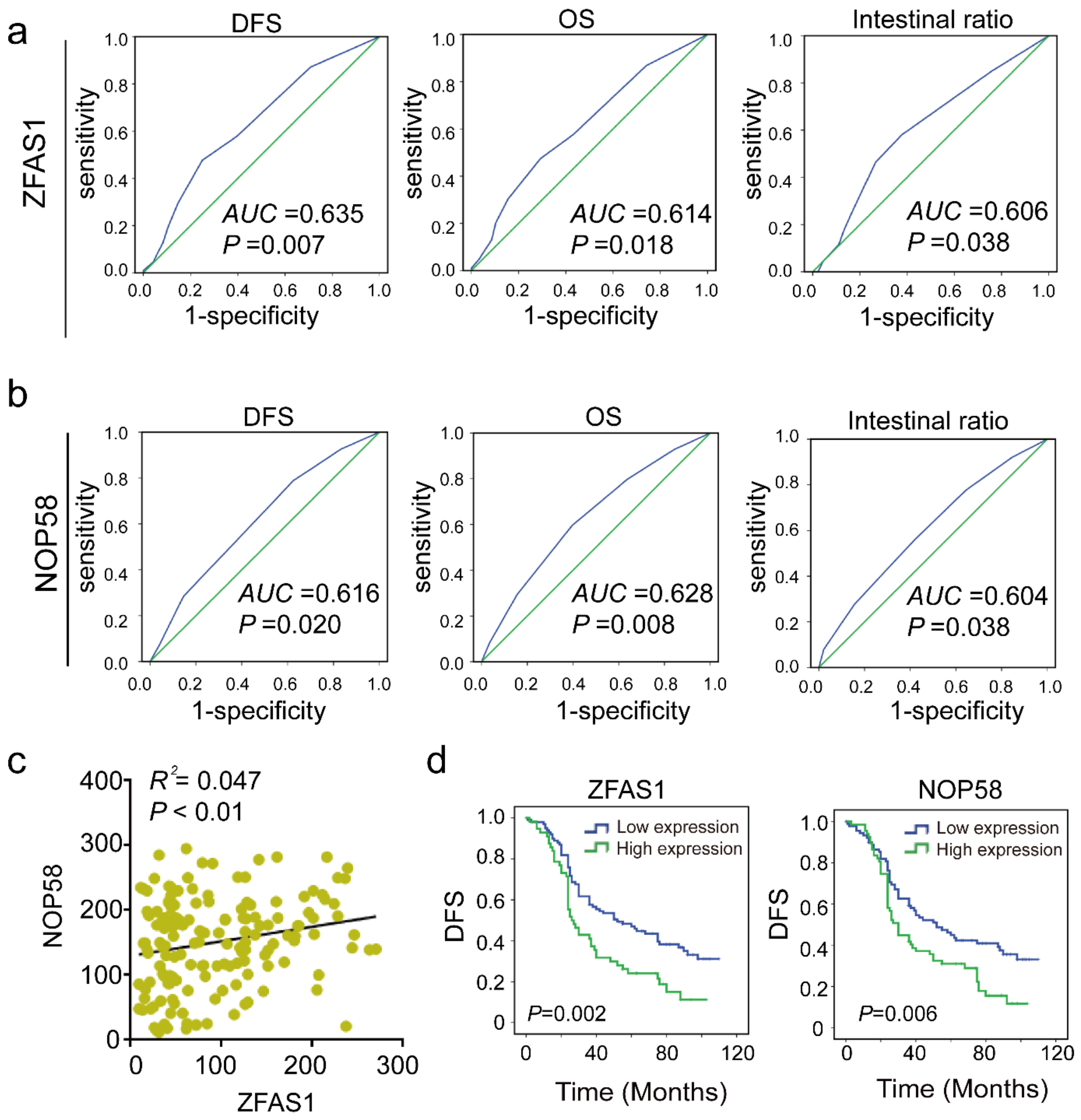

Fig. S5 The correlation of ZFAS1 and NOP58 expression and prognosis evaluation in CRC patient tissues

a, The ROC curves determining the cut-off values of ZFAS1 expression in paired CRC patient tissues vs. adjacent-tumor control tissues (*n*=157). **b**, ROC curves evaluating the cutoff values of NOP58 expression in paired CRC patient tissues vs. adjacent-tumor control tissues (*n*=157). **c**, The linear correlation analysis representing the relationship of the ZFAS1 expression and NOP58 expression. **d**, Kaplan-Meier curves representing the impact of ZFAS1 and NOP58 on disease-free survival in the CRC cohort (*n*=157).

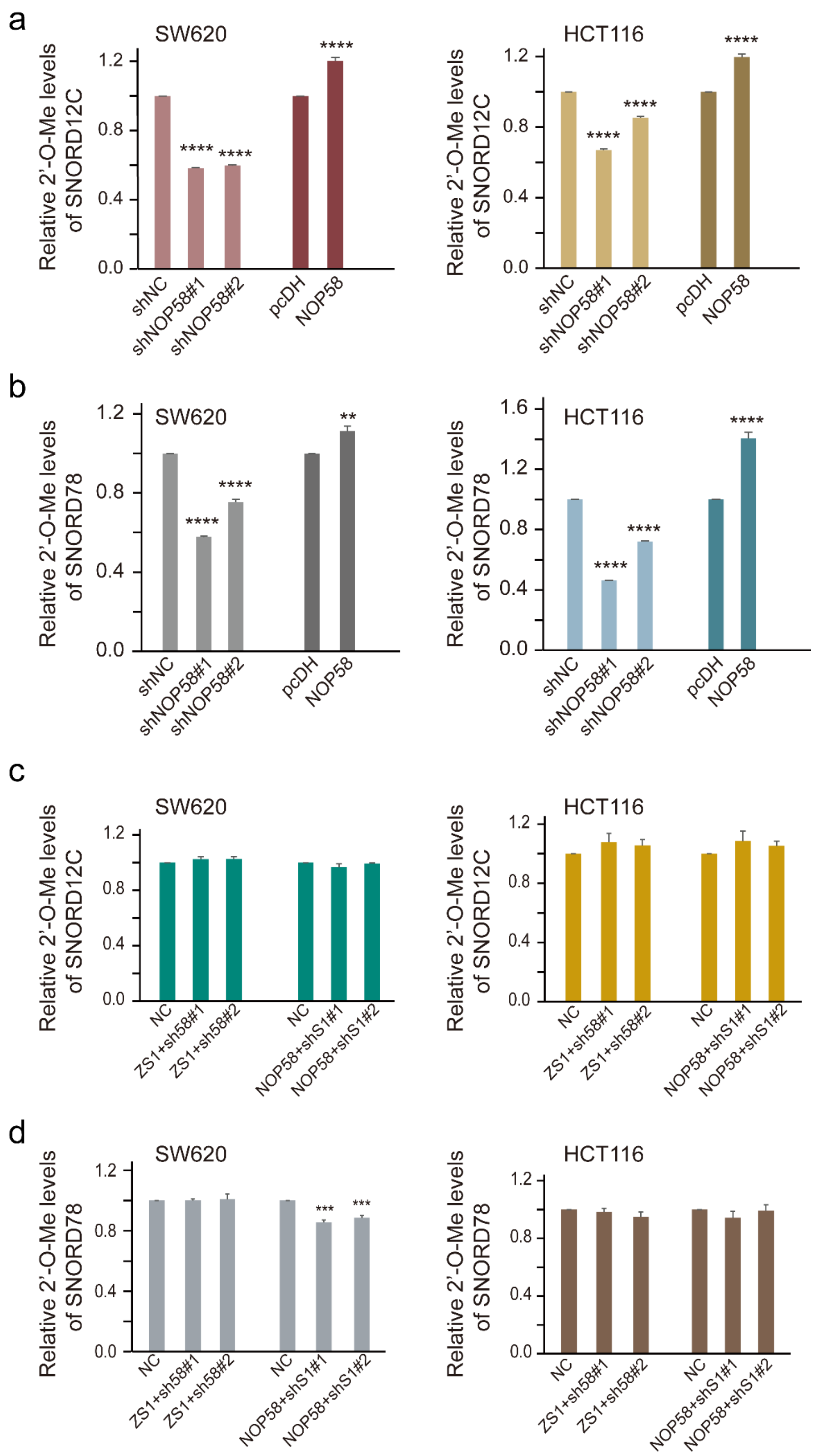

Fig. S6 The 2'-O-Me activity levels after interfering ZFAS1 and/or NOP58 expression
a, b, The histogram showing the 2'-O-Me activities mediated by SNORD12C (a) or SNORD78 (b) after silencing or overexpression of NOP58 in SW620 and HCT116 cells by RTL-P assay assays. **c, d**, The bar chart illustrating the 2'-O-Me activities mediated by SNORD12C (c) or SNORD78 (d) after co-transfection with pcDH-ZFAS1 and shRNA-NOP58 vectors in both SW620 and HCT116 cells by RTL-P assay.

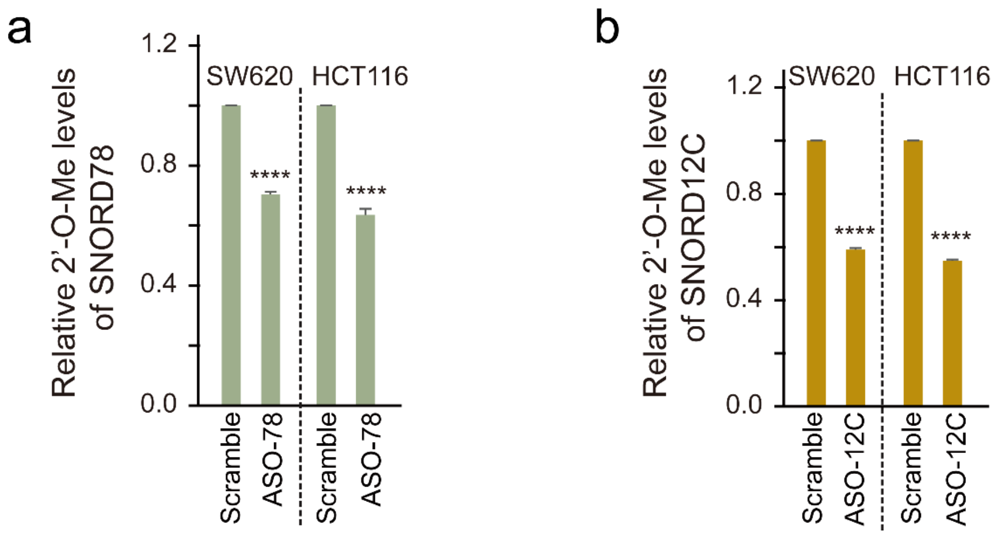

Fig. S7 The 2'-O-Me activities mediated by SNORD12C and SNORD78 after silencing SNORD12C and SNORD78

a, The histogram representing the 2'-O-Me activities after silencing SNORD78 (a) or SNORD12C (b) in SW620 and HCT116 cells assayed by RTL-P method.
